# Genomic and transcriptomic analyses reveal a tandem amplification unit of 11 genes and mutations in mismatch repair genes in methotrexate-resistant HT-29 cells

**DOI:** 10.1038/s12276-021-00668-x

**Published:** 2021-09-14

**Authors:** Ahreum Kim, Jong-Yeon Shin, Jeong-Sun Seo

**Affiliations:** 1 Asian Genome Institute, Seoul National University Bundang Hospital, Seongnamsi, 13605 Korea; 2 Department of Biomedical Sciences, Seoul National University College of Medicine, Seoul, 03080 Republic of Korea; 3 CHA University School of Medicine, Seongnam, South Korea; 4 Macrogen Inc., Seoul, 08511 Korea

**Keywords:** Cancer genomics, Genome evolution

## Abstract

*DHFR* gene amplification is commonly present in methotrexate (MTX)-resistant colon cancer cells and acute lymphoblastic leukemia. In this study, we proposed an integrative framework to characterize the amplified region by using a combination of single-molecule real-time sequencing, next-generation optical mapping, and chromosome conformation capture (Hi-C). We identified an amplification unit spanning 11 genes, from the *DHFR* gene to the *ATP6AP1L* gene position, with high adjusted interaction frequencies on chromosome 5 (~2.2 Mbp) and a twenty-fold tandemly amplified region, and novel inversions at the start and end positions of the amplified region as well as frameshift insertions in most of the *MSH* and *MLH* genes were detected. These mutations might stimulate chromosomal breakage and cause the dysregulation of mismatch repair. Characterizing the tandem gene-amplified unit may be critical for identifying the mechanisms that trigger genomic rearrangements. These findings may provide new insight into the mechanisms underlying the amplification process and the evolution of drug resistance.

## Cancer chemotherapy: Sequencing surplus gene copies

Sequencing a large region of DNA containing many surplus copies of genes linked to drug resistance in colon cancer cells may illuminate how these genomic rearrangements arise. Such regions of gene amplification are highly repetitive, making them impossible to sequence using ordinary methods, and little is known about how they are generated. Using advanced methods, Jeong-Sun Seo at Seoul National University Bundang Hospital in South Korea and co-workers sequenced a region of gene amplification in colon cancer cells. The amplified region was approximately 20 times the length of that in healthy cells and contained many copies of an eleven-gene segment, including a gene implicated in drug resistance. The region also contained mutations in chromosomal repair genes which would disrupt repair pathways. These results illuminate the genetic changes that lead to gene amplification and drug resistance in cancer cells.

## Introduction

Gene amplification, the triggering of an abnormal copy number increase in a specific region of the genome of cells growing under a selective condition, is associated with the overexpression of oncogenes such as *MYC, MYCN*, and *ERBB*, which engender abnormal cell proliferation and replication^1–3^. These genes undergo amplification much more frequently than simple mutation, since the rate of gene amplification is greater than the mutation rate in mammalian cells^4^; thus, such amplification has great tumorigenic potential^5^.

However, no molecular targeted agents have been specifically developed to prevent gene amplification because of the associated chromosomal complexity and technical limitations. It is therefore critical to find predictive and prognostic biomarkers for gene amplification to develop specific treatments that will improve patient outcomes and optimize therapeutic decisions^6,7^.

In addition, gene amplification is an indicator of a drug-resistant sample in cancer and healthy cells^8^, so it will be important to identify the genetic features or pathways that promote amplification in tumors. These mechanisms might serve as therapeutic targets that can prevent drug resistance and arrest or eradicate tumor cells^9^. However, the molecular mechanisms that contribute to high gene copy numbers are completely unknown, since sequence alignment and assembly programs that rely on short reads are not equipped to address genomic rearrangements and repetitive sequences^10,11^.

Short reads, which cannot be used to sequence repetitive sites, can only rarely be aligned to the accurate site of reference or the site of an abrupt change in copy number in the gene-amplified region, and previous research findings have proven the poor performance of short-read sequencing for structural variant detection as well as alignment^12,13^.

*DHFR* gene amplification at chromosome 5 is a hallmark of methotrexate (MTX) resistance in colon cancer cells and acute lymphoblastic leukemia. MTX is an antifolate drug that inhibits dihydrofolate reductase (*DHFR*) by preventing DNA synthesis and cell division^14–16^.

Amplification of the *DHFR* gene generates two major DNA segments consisting of extrachromosomal double minutes (DMs) and intrachromosomal homogeneously staining chromosome regions (HSRs); however, the molecular mechanisms underlying the generation of these amplified region products and suitable methods for their detection and characterization are still unknown.

In this study, single-molecule real-time (PacBio SMRT) sequencing, optical genome mapping (BioNano Genomics; read size: ~10 kb), and high-throughput chromosome conformation capture (Hi-C) for inter- and intrachromosomal interactions were used to identify relevant repetitive rearrangements with amplified segments and interpret gene amplification mechanisms in an MTX-resistant colon cancer cell line (HT-29), which has a heterogeneously amplified genome.

These techniques allowed the accurate quantification of amplification size and identification of drastic differences in chromosomal abnormalities and structural variants in MTX-resistant compared to MTX-sensitive samples, which are difficult to analyze in detail using short-read sequencing and fluorescence in situ hybridization (FISH). Accordingly, this study contributes to our understanding of basic genomic principles, the impacts of genetic rearrangements on cancer cells, and, by extension, how drug resistance arises from such rearrangements.

## Materials and methods

### Preparation of MTX-resistant cancer cells and sensitization procedure

After curating a list of cancer cell lines published in previous studies, we targeted human colon adenocarcinoma HT-29 cells. The HT-29 line was obtained from the Korean Cell Line Bank (KLCL) and cultured to develop MTX-resistant cancer cells, as described previously^17^. HT-29 was chosen because it can be engineered to grow in high concentrations of MTX via the amplification of *DHFR*.

Experimental and computational analyses were performed on a methotrexate-resistant colon cancer cell line (HT-29) to characterize the amplified gene region and understand the underlying mechanism (Supplementary Fig. 1).

To generate MTX-resistant HT-29 cells, we optimized the MTX concentration and cultured the cells in RPMI media supplemented with 10% fetal bovine serum and graded concentrations (from 10^−8^ to 10^−6^ M) of MTX in five T25 flasks per cell line. The MTX solution was prepared by mixing 100 mg MTX powder with 1.967 ml DMSO. This mixture was then aliquoted at 100 μl/ml and kept at −20 °C. A limited number of resistant cells (~3 × 10^5^) from each T25 flask were plated in Petri dishes in the same culture medium and MTX concentration.

We then used 3.2 mm-diameter cloning discs (Sigma) to transfer 3 isolated clones (C1–2, C4–3, C8–22) from each Petri dish to another T25 flask and then cultured these cells under the same conditions. Subsequently, the MTX-resistant clones were passaged 40 times without MTX. For the second and third treatment cycles, 3 × 10^5^ clones and parental HT-29 cells were cultured in 25 cm^2^ flasks. A stepwise increase in MTX concentration from 10^−8^ mol/L to 10^−6^ mol/L was applied; MTX-resistant cells were grown in 10^−6^ mol/L MTX.

### Detection of gene amplification

The copy number of MTX-resistant clones was measured via TaqMan Copy Number Reference Assays and Viia7 technology to select clones with high *DHFR* gene amplification. The *DHFR* gene copy number was estimated via relative quantitation (RQ) using the comparative Ct method and computed using the Ct difference (delta Ct) between MTX-resistant samples and reference samples (HapMap NA19982). Then, the ∆Ct values of MTX-resistant samples were compared to those of a HapMap reference sample known to have two copies of *DHFR*, such that they were two times the relative quantity of the reference. The following three equations were used to compute the copy number from the Ct value. The statistical significance was computed by the R package ‘pcr’) as described in the package instructions for MTX-resistant HT-29 samples.

1 $${\mathrm{Expression}} = 2^ - \left( {\left( {\mathrm{Sample}\;Ct - {\Re} \mathrm{ference}\;Ct} \right) - \left( {\mathrm{Control}\;Ct - {\Re} \mathrm{ference}\;Ct} \right)} \right)$$

2 $${\mathrm{Rate}}\;\mathrm{between}\;\mathrm{reference}\;\mathrm{and}\;\mathrm{sample} = \frac{{\mathrm{Expression}\;\mathrm{for}\;\mathrm{Sample}}}{{\mathrm{Expression}\;\mathrm{for}\;\mathrm{Reference}}}$$

3 $${\mathrm{Copy}}\;\mathrm{number} = 2\;\mathrm{copies}\;(\mathrm{reference}) \times \mathrm{Rate}$$

Samples with a high *DHFR* copy number were selected for FISH and karyotyping to visualize and map *DHFR* and detect chromosomal abnormalities. The *DHFR* red signal and 5p12-green signal were counted at anaphase and metaphase, with ×1000 magnification and a three-color (RGB) filter.

### RNA-seq analysis and transcriptome profiling

RNA-seq of MTX-resistant HT-29 cells and control samples was performed to investigate expression profiling. Total RNA, with ribosomal RNA removed, was prepared and sequenced using the Illumina HiSeq 2000 system (depth 100X), as previously described^18^. The obtained reads were mapped to the human reference genome (GRCh38) using the Spliced Transcripts Alignment to a Reference (STAR) tool to produce analysis-ready BAM files (Supplementary Table 1). We followed the key principles of processing and analysis steps from the GATK website. The mapped reads (BAM file) were visualized via SeqMonk.

To estimate the expression of each gene, raw reads were counted using the HTSeq-count tool and normalized to variance stabilizing data (VSD) expression via the R package DEseq2. Fragments per kilobase million (FPKM) values were calculated using the R package edgeR and converted to log_2_ values. The median-centered gene expression was computed from FPKM expression using Cluster 3.0 software, which subtracts the row-wise median from the expression values in each row. The median-centered VSD and FPKM expressions were visualized in a heatmap.

### Variant discovery analysis

Variant calling was performed on transcriptome datasets. The duplicated sites from analysis-ready BAM files were filtered with Picard, and variants were called and filtered by removing spurious and known RNA-editing sites in VCF format. The genomic variants in the control and MTX-resistant HT-29 samples were compared. We performed variant discovery analyses according to the step-by-step recommendations provided by the Genome Analysis Toolkit (GATK) to obtain high-quality variants^19^.

To determine the exact SNPs from the call set, the variants were filtered out according to several criteria. First, the cutoff for quality-by-depth (QD) was 3.0, which is the variant confidence score divided by the unfiltered depth of coverage. Variants were filtered out if they had a score <3.0. Second, the variants were filtered out when the Fisher strand (FS) score was >30.0, which indicated the Phred-scaled *p*-value using Fisher’s exact test for detecting strand bias^20^. The identified and filtered variants were annotated using RefSeq genes and the ANNOVAR tool.

### Differentially expressed gene analysis

Differentially expressed genes (DEGs) between MTX-resistant HT-29 cells and control samples were analyzed using the R packages DESeq2 and edgeR (*P*-value < 0.05, |Log2 (fold change)| ≥ 1, and baseMean ≥ 100). The DEGs were then subjected to enrichment analysis with KEGG gene sets via Gene Set Enrichment Analysis (GSEA).

### Alternative splicing event analysis

The exon inclusion levels, defined with junction reads from RNA sequencing results, were subsequently processed via rMATS.3.2.5^21^. Five different types of alternative splicing events (SE: skipped exon, MXE: mutually exclusive exon, A5SS: alternative 5′ splice site, A3SS: alternative 3′ splice site, RI: retained intron) were identified in both MTX-resistant HT-29 cells and control samples. The number of significant events was detected using both junction counts and on-target reads.

### PacBio long-read sequencing analysis

Genomic DNA was extracted from MTX-resistant HT-29 cells and control samples using the Gentra Puregene Cell kit (Qiagen), and libraries were constructed for PacBio sequencing. The PacBio long reads were aligned to the human genome (version GRCh38) with BWA-mem aligner, and the preprocessing pipeline on the BWA-mem website was followed to ensure the technical and biological quality of the results (Supplementary Table 2). The read depth was estimated via the depth-of-coverage option in GenomeAnalysisTK. Regions with coverage differences of >10X between MTX-resistant HT-29 cells and controls were selected as amplified regions.

### Detection of genomic variants and amplification units

The structural variants (deletion, duplication, inverted duplication, translocation, and inversion) in MTX-resistant HT-29 cells and control samples were analyzed from sorted PacBio output derived from BWA-MEM using Sniffles^22^. The BAM files were converted to binned copy numbers across a genome using Copycat. The genomic rearrangements were visualized from VCF files (Sniffles) and read coverage files (Copycat) via SplitThreader (http://splitthreader.com/)^12^. BWA-MEM and Sniffles were used in combination to successively scan the alignments to identify all types of SVs in tandem gene-amplified regions, including repeat-rich regions and complex nested events^23^.

### Long-read sequencing and optical genome mapping

The long-read sequencing coverage was designed to be ~10X, and the coverage was not sufficient to perform de novo assembly except in the tandemly amplified region (~197X). Thus, only the 54,804 PacBio reads from our tandemly amplified region were de novo assembled using the PBcR assembler, and 10 contigs were generated by using the previously reported method^24^. Of these, 6 contigs were used for reference-assisted genome ordering utility (Ragout) with hg 38, and we had one scaffold from 6 contigs. The other contigs were alternatively assembled and finally matched with other contigs. In addition, PacBio PBcR contigs were compared with the Bionano contigs to identify the accuracy of assembly (Supplementary Tables 3 and 4).

Optical mapping of the PacBio assembly data was performed using BioNano Assembler software (Irys System, BioNano Genomics) to obtain accurate sequences. High molecular weight DNA was isolated using the IrysPrep Plug Lysis Long DNA Isolation Protocol (Bionano Genomics). In brief, cells were trypsinized, washed in FBS/PBS, counted, rewashed in PBS, and embedded in agarose plugs using components from the Bio-Rad Plug Lysis Kit. The plugs were subjected to proteinase K digestion (2 × 2 h at RT). After a series of washes in the buffer from the Bio-Rad kit, followed by washes in TE (Tris-EDTA), the plugs were melted and treated with GELase enzyme (Epicenter).

High molecular weight DNA was released and subjected to drop dialysis. The DNA was left to equilibrate for 4 days and then quantified using the Qubit Broad Range dsDNA Assay Kit (Thermo Fisher Scientific). Using the IrysPrep NLRS assay (Bionano Genomics), 200–300 ng/µL of high molecular weight DNA underwent single-strand nicking with 10 units of Nt.BspQI nickase (New England BioLabs). Nicked sites were repaired with fluorophore-labeled nucleotides to restore strand integrity.

The backbone of the fluorescently labeled double-stranded DNA was stained with the intercalation dye YOYO-1. Labeled molecules were loaded directly onto IrysChip without further fragmentation or amplification and imaged using the Irys instrument. Multiple cycles were performed to reach an average raw genome depth-of-coverage of 50X. In addition, the tandem repeats in the amplified region in both the assembly and raw data were identified using IrysView 2.0 software (Supplementary Tables 5 and 6).

### Hi-C data analysis

Approximately 50 million MTX-resistant HT-29 cells and control cells were used to produce high-throughput chromatin conformation capture (Hi-C) datasets. We generated 2 Hi-C libraries using the HindIII restriction enzyme, following a previously established protocol^25^. In brief, the Hi-C protocol involves crosslinking cells with formaldehyde, permeabilizing them while keeping the nuclei intact, digesting the DNA with a suitable restriction enzyme, filling the 5′-overhangs while incorporating a biotinylated nucleotide, ligating the resulting blunt-end fragments, shearing the DNA, capturing the biotinylated ligation junctions with streptavidin beads, and analyzing the resulting fragments with paired-end sequencing via Hi-Seq2000.

The HiC data (fastq files) were processed to normalize the contact matrices using HiC-Pro version 2.10.0^26^. The pipeline was based on the Bowtie 2 aligner, and the selected restriction enzyme (HindIII) was used to generate normalized contact maps, as described in the HiC-Pro pipeline (https://github.com/nservant/HiC-Pro). Each aligned read was assessed to determine the valid interactions and control quality by excluding invalid ligation products and duplicated valid pairs (Supplementary Table 7). The aligned Hi-C sam files were converted into the HiCnv format, which calls CNVs from Hi-C data^27^. In addition, interchromosomal translocations and their boundaries were detected from a Hi-C matrix file using HiCtrans. The list of valid interaction output files called by HiC-pro was converted to a Juicebox input file and visualized using Juicebox (https://github.com/theaidenlab/juicebox/wiki).

The R package HiCcompare was used to detect differential spatial chromatic interactions on a genome-wide scale between control and MTX-resistant HT-29 cells^28^. Using this package, the interaction frequencies after adjustment with joint-normalization, adjusted *p*-values, and filtered low-average expression after multiple testing correction were determined.

To remove background interactions from the inter- and intrachromosomal interactions, we compared chromosomal interactions between the control and MTX-resistant samples by considering the combination of eigenvector, balanced value, and coverage, as well as the interaction frequencies after adjustment with joint normalization. Hi-C contact maps were visualized in Juicebox with applied balanced normalization and eigenvector and coverage. We calculated the eigenvector (first principal component) of the Pearson correlation of the (binned) HiC contacts and annotated contact domains by using the arrowhead option, and balanced normalization (Knight-Ruiz) was used to remove background.

### Statistical tests

All statistical analyses were performed using R-3.3.0. The gene expression levels in MTX-resistant and control HT-29 cells were compared, and the *p*-value was determined using unpaired Student’s *t*-test or Mann–Whitney *U*-test, based on the Shapiro–Wilk normality test. *P*-values <0.05 were considered to be statistically significant.

### Transposable element detection

Transposable elements (TEs) were identified and compared between control and MTX-resistant HT-29 cells by using Censor, which is a program for the detection of TEs^29^.

## Results

### Analysis of MTX-resistant HT-29 cells

While generating MTX-resistant HT-29 cells (selected clone: C1-2) from single-cell selection and MTX sensitization as previously described^30^, dramatic morphological changes in the cells themselves were observed; rounded and circular cell shapes were observed during the first cycle of sensitization, and rod and irregular shapes were seen during the second cycle (Supplementary Fig. 2). The original shapes were again observed during the third cycle of sensitization, which might indicate that HT-29 cells became resistant to MTX and grew rapidly under high MTX concentrations.

As expected, *DHFR* expression, which was normalized to the expression of the *B2M* housekeeping gene, steadily increased from the first to third cycle, as the HT-29 clones became resistant to MTX (Supplementary Fig. 3a). After confirming these morphological changes and increased *DHFR* expression, the *DHFR* copy number was measured in several clones.

The cycle quantification values (Ct) of the clones were used to compute the rate of copy number change. These values dropped from 26.04 to 19.99 as the number of cycles increased (estimate: 4.075, *p* = 0.003758396, [95% CI]=1.8–6.3, Supplementary Table 8). Interestingly, there was a dramatic increase in the *DHFR* copy number between the first (0.97 copies) and second (54.83 copies) cycles (Supplementary Fig. 3b).

Based on these results, we ascertained that a specific time period and set of conditions were required for clones to survive in the presence of MTX, and amplification of the *DHFR* gene was an indicator of MTX resistance, as previously described^14^.

### Validation of gene amplification in MTX-resistant HT-29 cells

After quantifying the *DHFR* gene copy number, the amplification of the *DHFR* gene at the 5q arm was visualized via fluorescent in situ hybridization (FISH) of an MTX-resistant clone (C1-2) and control sample. The *DHFR* gene regions on the 5q arm of cells in the second and third cycles were abnormally long compared to that of the control, as expected (Supplementary Fig. 4).

The amplified *DHFR* gene patterns of C1-2 cells in metaphase revealed that the amplified region had a chromosome painting signal, and the FISH signal patterns were highly heterogeneous. In total, 9 minor signal-amplified patterns and two major patterns accounted for 44 and 28% of abnormal *DHFR* gene amplification patterns, respectively (Supplementary Fig. 5).

**Fig. 1:**
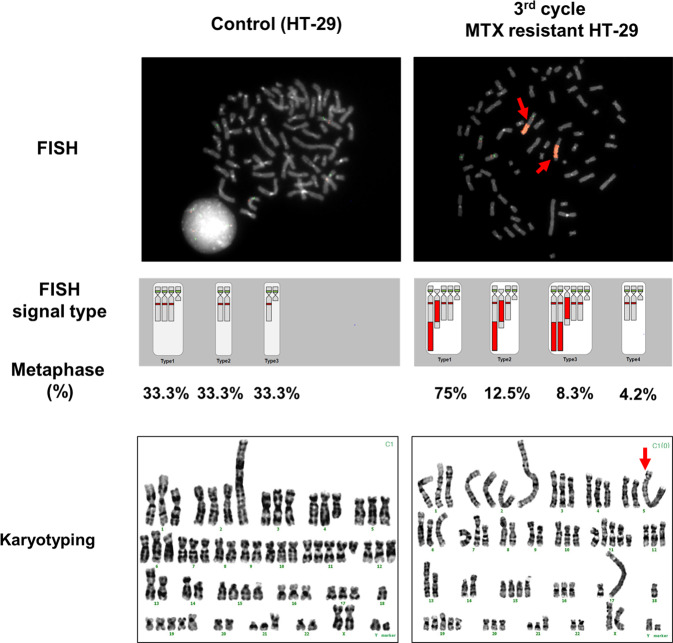
Visualization of *DHFR* amplification patterns using FISH and karyotyping of MTX-resistant clones (C1-2-4) and controls. A subculture (C1-2-4) of the C1-2 clone was investigated via fluorescent in situ hybridization (FISH) at ×1000 magnification, and the FISH signal type and percentage at metaphase were compared between controls and MTX-resistant clones (C-1-2-4). All chromosomes were karyotyped, and abnormal chromosomal shapes were detected for the 5q and 17q arms of MTX-resistant cells compared to controls, as indicated by the red arrow.

The optimized C1-2 clone (C1-2-4), which was robust in the presence of MTX, was visualized using FISH. Four different types of gene amplification patterns were detected: two patterns had amplified *DHFR* genes at two q arms (75 and 12.5%), one pattern had amplified DHFR genes at three q arms (i.e., an isochromosome pattern) (8.3%), and one completely lacked amplified *DHFR* genes at q arms (4.2%) (Fig. 1). Overall, ~96% of the cells had at least one *DHFR*-amplified region.

A majority of these strains were resistant to MTX and exhibited a high level of *DHFR* gene amplification at the 5q arm. However, the MTX-resistant C1-2-4 cells had a variety of *DHFR* gene amplification patterns and copy numbers, as well as heterogeneous genetic status in the presence of MTX, even though the strain was generated from a single MTX-resistant cell^31^.

In addition, MTX-resistant HT-29 and control samples were karyotyped to accurately detect the amplified region. The C1-2-4 clone had a homogeneously stained region (HSR) at the chromosome 5q arm, as previously described^32^, in addition to an abnormally long 17q arm (Fig. 1). It was previously found that chromosome 17q arm amplification could occur because of the overall genetic instability and karyotype diversity in colorectal cancer^33^.

### Analysis of the structural variants and amplification units

**Fig. 2:**
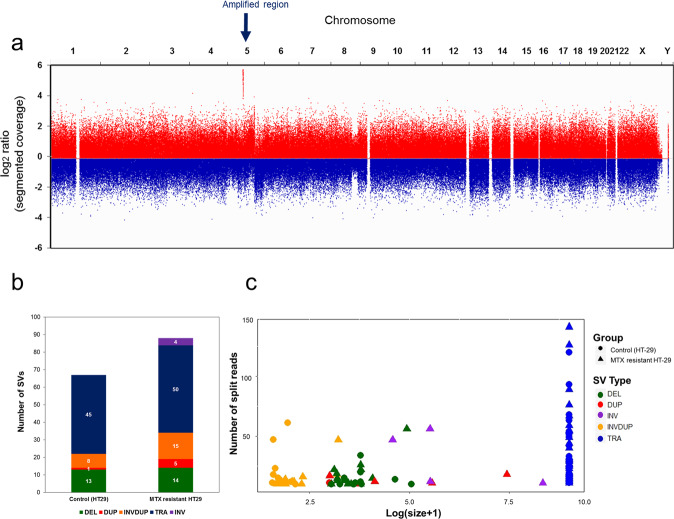
Detection and characterization of SVs in MTX-resistant HT-29 cells. **a** The log2 ratio of segment coverage across all chromosomes was compared between control and MTX-resistant HT-29 cells. The amplified region on 5q in MTX-resistant HT-29 cells is indicated by the blue arrow. **b** Five genomic variants (deletion, duplication, inverted duplication, translocation, and inversion) in the MTX-resistant sample and control were analyzed and visualized in bar graphs, which show the number of variants counted for each sample. **c** In the scatter plot, the size of variants (log(size+1)) and depth of split reads were plotted for control and MTX-resistant HT 29 cells. The group and variant type are indicated by a different shapes and colors.

After confirming the HSR on the amplified *DHFR* region, the five genomic structural variants (deletion, duplication, inverted duplication, translocation, and inversion) and amplified units of MTX-resistant cells were analyzed to identify which genes and structural variants were involved in gene amplification. The log_2_ ratio of segment coverage over whole chromosomes between the control and MTX-resistant samples was compared. High segment coverage in the MTX-resistant sample, compared to that of the control sample, was observed on chromosome 5 (Fig. 2a). The genes for which the segment coverage was larger than 20 were identified and annotated by position to identify the exact amplified region that included the *DHFR* gene.

The total number of genomic variants in the MTX-resistant sample was larger than that in the control, and the size of variants such as duplicates and inversions was larger in the MTX-resistant sample, which had a large number of split reads (Fig. 2b, c). In addition, more structural variants were detected in MTX-resistant HT-29 cells than in control cells (Supplementary Data 1 and 2).

Selected novel structural variants on chromosome 5 were compared between control and MTX-resistant HT-29 cells. One duplication (median size of split reads: 371,675), three inversions (median size of split reads: 328,697), and three inverted duplications (median size of split reads: 1529) were detected in the MTX-resistant HT-29 cells, while no such variants were detected in the control sample (Table 1). Moreover, the number of split reads for the MTX-resistant HT-29 cells was larger than the average coverage (10X), and most copy number variation (CNV) categories matched, which indicated that the detected structural variants had been accurately detected (Supplementary Table 9 and Supplementary Data 3). Finally, although the number of translocations on chromosome 5 was decreased in the MTX-resistant sample, there were more detected interchromosomal genomic rearrangements in the MTX-resistant HT-29 sample (Supplementary Fig. 6).

**Table 1:** Comparison of detected structural variants between control and MTX-resistant HT-29.

No. of events (median size)	All chromosomes		Chromosome 5	
	Control (HT-29)	MTX-resistant HT-29	Control (HT-29)	MTX-resistant HT-29
Deletion	13 (4472)	14 (2917)	2 (4425.5)	3 (1514)
Duplication	1 (1004)	5 (13534)	0	1 (371675)
Inversion	0	4 (331155.5)	0	3 (328697)
Inverted duplication	8 (47)	15 (77)	0	3 (1529)
Translocation	45	50	4 (chr3 to chr5) and (chr5 to chr12)	2 (chr5 to chr12)

The structural variants (deletions, duplications, inversions, inverted duplications, and translocations) were categorized by using Sniffles from sorted PacBio output derived from BWA-MEM over all chromosomes and chromosome 5 in control (HT-29) and MTX-resistant HT-29 cells. The median size of split reads is shown in parentheses.

**Fig. 3:**
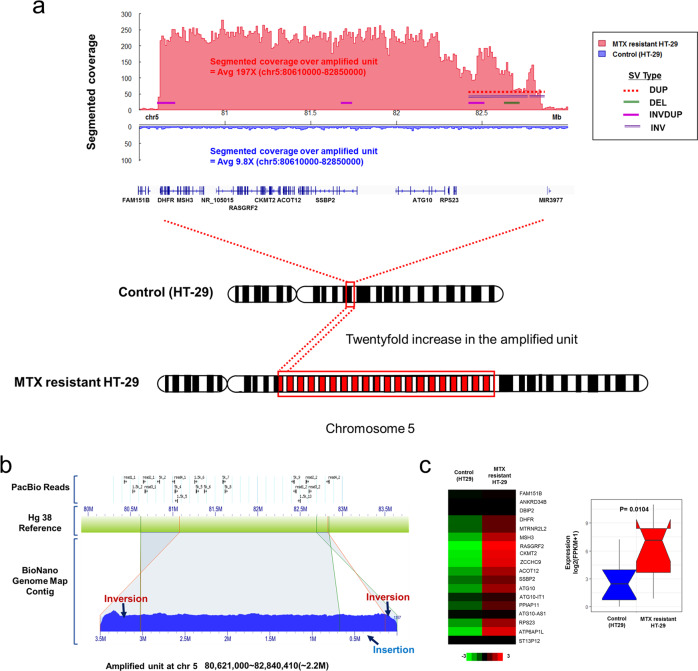
Detection of amplified units and structural variants over the amplified region in MTX-resistant HT-29 cells. **a** The segment coverage and genomic variants across the amplified region on chromosome 5 (80,610,000-82,850,000) were compared between control and MTX-resistant HT-29 cells. **b** The BioNano contig and PacBio long reads matched up with the reference (hg38); several genomic rearrangements are indicated by blue arrows. **c** The heatmap depicts the gene expression (FPKM) from the amplified units for MTX-resistant HT-29 cells and controls. High expression in a specific region is indicated by a red arrow. The statistical significance (*p*-value) was computed using the Mann–Whitney test.

The position of the amplified region on the chromosome 5q arm was between 80 Mb and 83 Mb, which involved the *DHFR* gene as the start point and included the *ATP6AP1L* gene. The segment coverage was ~197X, between 80.6 Mb and 82.8 Mb (~2.2 Mb) (Fig. 3a and Supplementary Fig. 7). The amplified region was ~20-fold longer in the MTX-resistant sample than in the control. This size was inferred from the long-read sequencing coverage, which was 10X in the control sample but abnormally high (~197X) in only this region in the MTX-resistant sample.

Overall, the amplification regions included tandem gene amplifications of 11 genes in this region, from *DHFR* to *ATP6AP1L*, and involved inversions or inverted duplications at the end of the amplification unit. The tandem duplications of several genes on chr5 (2.2 Mbp) were initiated and terminated by inversion on the specific sequence. The inversion identified at the start point (chr5: 80,618,750-80,631,490) of the amplification unit was evidenced by the three-dot plots compared to hg38. The inverted region included four genes, *LINC01337, DHFR, CTC-325J23.2*, and *MTRNR2L2*, and the identified inversion was confirmed by PacBio alignment; however, but other structural variants were not detected because of the difficulty and poor performance of assembly (Supplementary Figs. 8 and 9).

### Optical genome mapping over the amplified region

The region of tandem amplification (80.6–82. Mbp) on chromosome 5 was further analyzed using BioNano genome optical mapping, as good coverage and mapping over the whole range of the amplified region could not be achieved using other methods. The genome mapping contigs mapped in complex ways to whole chromosomes, except for the amplified region, for which there was high coverage of ~200X (Supplementary Fig. 10).In addition, the gene-amplified unit had inversions at both the start and endpoints of the amplified region, as expected, and there was a newly identified insertion at the endpoint of the amplified region (Fig. 3b).

These inversions were certainly associated with the amplification mechanism and seemed to assist with and even initiate tandem repeat amplification, as previously reported^34^. The identified inverted repeat could stimulate the formation of a large DNA palindrome after the breakage of an adjacent DNA double-strand^35^.

### Gene expression levels in the MTX-resistant sample

There was extremely high gene expression within the identified amplification region, consistent with the high coverage of the amplified unit in our long-read sequencing data (Supplementary Fig. 11 and Supplementary Table 10). The read coverage from long-read sequencing of the amplified region in the MTX-resistant sample was ~10 times higher than that of the control. Similarly, the log_2_(FPKM + 1) expression level from the *DHFR* to *ATP6AP1L* gene in MTX-resistant HT-29 cells was 5X that of the control for *DHFR* and 122X that of the control for *RASGRF2* (*P* = 0.0104 via the Mann–Whitney test; Fig. 3c).

### Identifying novel mutations and their impacts on gene amplification

To identify relevant mutations in the amplification mechanism, single-nucleotide variants (SNVs) in both samples were identified using transcriptome sequencing data. There were more total exonic mutations across all chromosomes in MTX-resistant HT-29 cells (13,982) than in the control sample (13,310), and on chromosome 5 specifically, 18 more exonic mutations were detected in MTX-resistant HT-29 cells than in controls (Supplementary Tables 11 and 12 and Supplementary Data 4 and 5).

After filtering out synonymous SNVs, we observed that there were several more nonsynonymous mutations (nonsynonymous SNVs, frameshift deletions, frameshift insertions, stop-gains, and stop-losses) in MTX-resistant HT-29 cells compared with control cells across all chromosomes (Supplementary Fig. 12). Some nonsynonymous mutations came from chromosome 5, in which the number and percentage of frameshift insertions were noticeably higher in the MTX-resistant sample (18.1%) than in the control (4.3%) (Supplementary Fig. 4a). The most frequently inserted nucleotides in frameshift insertions were thymine (73%) and adenine (23%).

**Fig. 4:**
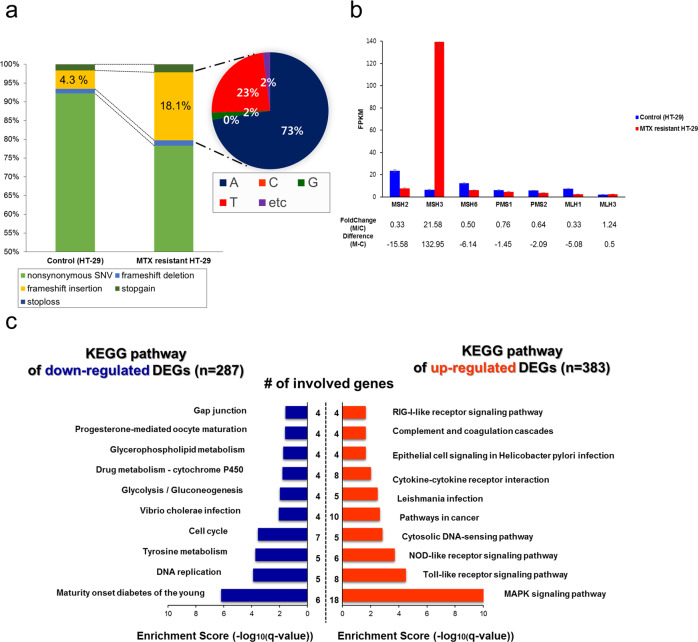
Comparison of nonsynonymous mutations and *MMR* gene expression levels. **a** Nonsynonymous mutations in chromosome 5 (nonsynonymous SNV, frameshift deletion, frameshift insertion, stop-gain, and stop-loss) were compared between MTX-resistant HT-29 cells and control cells. The proportions of the four nucleobases in the frameshift insertions in the MTX-resistant HT-29 cells are indicated. **b** Gene expression (FPKM) of mutS homologs and mutL homologs was computed and compared. The changes and differences between MTX-resistant HT-29 cells (M) and control cells (C) are indicated below the bar graph. **c** The top 10 enriched KEGG gene sets among upregulated and downregulated differentially expressed genes are shown according to enrichment score (−log(*q* value)).

This imbalance might explain why the detected frameshift thymine and adenine insertions in mRNA on chromosome 5 co-occurred with *DHFR* gene amplification, which conferred an ability to survive MTX exposure. Moreover, novel frameshift insertions—either adenine or thymine—were found within the *MSH3* and *MSH6* genes as well as the *PMS1* and *PMS2* genes in the MTX-resistant HT-29 sample only (Supplementary Table 13). The expression of these genes, except for *MSH3*, was decreased in the mutated and MTX-resistant HT-29 cells compared to the control sample (Fig. 4b).

The *MSH3* and *MSH6* genes belong to the mismatch repair (*MMR*) gene family and are known to play an important role in DNA repair during cell division as well as in cooperatively suppressing intestinal tumors^36^*. MLH* (*PMS1*) and *MSH* (*MSH1 - 6*) gene function is significantly correlated with colon cancer; mutations in these genes could cause predisposition and susceptibility to Lynch syndrome, in conjunction with colon cancer^37–39^.

Therefore, novel frameshift insertions in these genes could prevent mismatch repair functionality and tumor suppression in the presence of MTX and stimulate the rapid progression of gene amplification and MTX resistance. The molecular explanation for this tandem gene amplification mechanism could be a malfunction of the MMR pathways^40^. It was previously shown that *MSH3* is concurrently amplified with the *DHFR* gene in MTX-resistant cells due to their proximity to each other. An imbalance in the expression of mutS homologs could result in the malfunctioning of base-base mismatch repair and cause genetic instability as well as confer resistance to the cytotoxic effects of MTX^41^.

### Identifying DEGs

To determine which genes and gene sets are involved in MTX resistance, we analyzed DEGs and enriched gene sets (Fig. 4c). A total of 383 upregulated and 287 downregulated DEGs were identified in MTX-resistant HT-29 cells compared to the control sample (Supplementary Table 14).

KEGG enrichment analysis of gene sets was also performed. The DEGs upregulated in MTX-resistant HT-29 cells included *IL1B, MAPK11, JUN, MAP3K8, IL8*, and *CASP1*. The affected signaling pathways included MAPK, Toll-like receptor, and NOD-like receptor signaling. Interestingly, the downregulated DEGs, such as *MAD2L1, CCNA2, MCM2, MCM4, FEN1*, and *CDK6*, were enriched in DNA replication, tyrosine metabolism, and cell cycle pathways, which are commonly upregulated in colon cancer^42^.

It was previously reported that the DEGs downregulated in MTX-resistant osteosarcoma cell lines were enriched in the mitotic cell cycle, cell cycle, and DNA replication pathways, and these pathways were also downregulated in our MTX-resistant colon cancer cells. This result might explain the role of MTX in inhibiting *DHFR* and preventing tumor cells from proliferating in both cases^43,44^. In addition, downregulation of MMR gene expression, which affects G2/M cell cycle arrest and apoptosis, might prevent proper checkpoint and cell death signaling; therefore, this phenomenon could contribute to the malfunctioning of DNA replication^45^.

### Chromosomal interactions and topologically associating domains (TADs)

Genome-wide intrachromosomal interactions were identified in MTX-resistant HT-29 cells and controls at 5 kb resolution and compared (Supplementary Fig. 13). A high interaction frequency was apparent, with a clear long red line, on the amplified region only (5q14.1 to 5q14.2) in the MTX-resistant HT-29 cells. This interaction pattern was similar to those observed for amplification regions in tumor samples in a previous study^46^ (Supplementary Fig. 14).

**Fig. 5:**
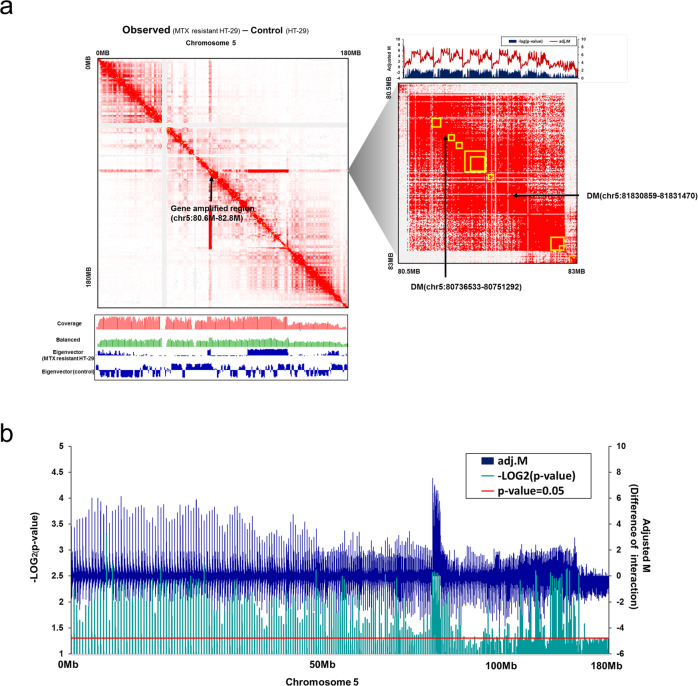
Topologically associating domains on chromosome 5 and adjusted interaction frequencies. **a** Intrachromosomal interactions for chromosome 5 (MTX-resistant HT-29 – control) are visualized with the coverage and eigenvectors (left), and the newly identified topologically associating domains (TADs) on the duplicated region are indicated by a yellow box at 500 kb resolution (right). **b** The difference in adjusted interaction frequencies (adjusted M) between MTX-resistant and control samples is plotted on a −log2 scale (*p*-value). Statistical significance (*p*-value = 0.05) is indicated by the red line.

The TADs and several chromosomal rearrangements at chromosome 5 were identified to visualize the conformations and intra- or interchromosomal interactions within the amplified region and to detect unforeseen chromosomal rearrangements at 500 kb resolution (Fig. 5a). Frequent intrachromosomal interactions were observed in the amplified region (chr5: 80.6–82.8 Mb), and there were several newly identified TADs in the middle and endpoint of this region compared to those of the control with high adjusted interaction frequencies (adjusted M) and an adjusted *p*-value < 0.05 (Supplementary Table 15).

Interestingly, interactions were also frequent within the region between 109 and 138 Mb, and more TADs and high eigenvector values were observed. These delineated compartments in Hi-C^47^ and indicated that both the amplified region and an adjacent region had both frequent intrachromosomal interactions and frequent contacts, and it seemed that the entire 5q region was upregulated by the amplification mechanism.

To compare the computed intrachromosomal interactions between MTX-resistant HT-29 cells and control samples, the difference in adjusted interaction frequencies (adjusted M) at 500 kb resolution and corresponding *p*-values were analyzed, and differentially interacting genomic regions at chromosome 5 were identified in the amplified region (*p*-value < 0.05; Fig. 5b). The adjusted M values were lower in the region from 109 to 138 Mb than in the amplified region, but this region still had a significant p-value and a higher level of interactions compared to those in other positions. Therefore, the chromosomal structure, from the start position of the amplified unit (80 Mb) to its endpoint on 5q, could involve a complex network of spatial contacts to accommodate the gene amplification.

The relative copy number of each chromosome was estimated from Hi-C data using chromosome 2 as a reference. Specifically, the relative copy number of chromosome 5 was significantly higher in MTX-resistant HT-29 cells than in control cells over all chromosomes except for chromosome 17, which also had a stretched structure similar to that observed for chromosome 5 (Supplementary Fig. 15).

In addition, we observed chromosomal rearrangements, such as those that would result in DMs of extrachromosomal DNA stretches that harbored amplified oncogenes that were involved in drug resistance. These rearrangements were detected in the amplified region via Hi-C and were not observed in the FISH data. Hi-C data were utilized because they could reveal unforeseen chromosomal rearrangements and CNV in highly amplified regions. However, this result should be confirmed using other techniques, since it was not clear how to distinguish between DMs and HSRs due to their similar structure.

### The mechanisms underlying tandem gene amplification

Using Censor, which identifies TEs, we analyzed the TEs, and we detected 46 TEs in the MTX-resistant sample (Supplementary Table 16)^29^. LTRs (43%, total insertion: 20), NonLTR/SINE/SINE1s, including Alu (8%, total insertions: 4), and NonLTR/L1s, including LINE-1 (13%, total insertions: 6), were detected. The detected retrotransposon insertions could promote structural instability, and Alu insertion in mismatch repair genes could affect recombination, deletions, and insertions throughout the genome, as previously reported^48^. In addition, LTRs (long terminal repeats) can alter alternative splicing patterns and polyadenylation signals. The L1ME3C_3 end of non-LTR/L1, which is a tandem gene repeat unit, was detected on *RASGFR2* of chromosome 5. However, it was not found at the site of a structural variant such as an inversion, which was the starting position of gene amplification. Therefore, we concluded that LINE-1 (L1ME3C_3end) was inserted into the *RASGFR2* gene but that this insertion was not the event that triggered chromosomal breakage^49,50^.

**Fig. 6:**
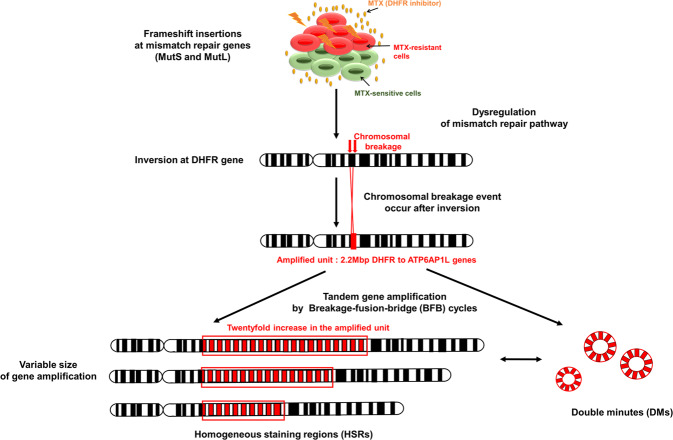
The mechanism of tandem gene amplification in the presence of MTX. Frameshift insertions in mismatch repair family genes (mutS and mutL homologs) can initiate the dysregulation of mismatch repair pathways. A chromosomal breakage event occurs after the emergence of an inversion at *DHFR*. Those breaks that occurred between *DHFR* and *ATP6AP1L* (2.2 Mb) were involved in producing the amplified unit. Finally, tandem amplifications of variable size can be achieved via breakage fusion-bridge (BFB) cycles.

We have developed experimental and computational workflows to detect and analyze the mechanisms underlying gene amplification in MTX-resistant HT-29 cells through breakage-fusion-bridge (BFB) cycles, as previously reported^51^ (Fig. 6). Before gene amplification occurred, frameshift insertions in *MSH* and *MLH* genes across several chromosomes were caused by MTX toxicity, which resulted in a genetic predisposition to gene amplification and dysregulation of mismatch repair pathways in the presence of MTX^52,53^.

After the malfunctioning of mismatch repair mechanisms in the presence of MTX, chromosomal breakages occurred at the *DHFR* gene on chromosome 5 because of the inverted repeat at the start position of the gene. In addition, inverted repeats from the *DHFR* gene to *ATP6AP1L* (2.2 Mb) were involved in producing the amplified unit. The endpoint of the amplified unit had the same inverted repeat, which could indicate the end position of the gene amplification. However, it is still unknown how the amplification of these specific genes was selected for and what factors are involved in the gene amplification mechanism.

Finally, variable sizes of amplified regions leading to HSRs could be produced via BFB cycles, and the unstable HSRs could be occasionally transformed into either a HSR fragment of a different size or DMs, accompanying inversions at the endpoints. Overall, the coamplification of *MSH3* and *DHFR* and frameshift mutations in *MSH* and *MLH* continuously affected genetic instability and enhanced resistance to methotrexate.

## Discussion

Recent developments in NGS technologies have enabled the generation of long reads (~10 kb) and are able to produce enormous amounts of genomic information at base-level resolution. Such detailed data can facilitate the analysis of uncharacterized regions that cannot be studied using other technologies^54,55^.

Here, complicated cancer genomes and abnormal chromosome structures were detected and analyzed using a combination of advanced technologies, including PacBio SMRT, optical mapping, and Hi-C analysis, as well as short-read sequencing and FISH. An integrative framework was also used to detect and interpret the structural variations in MTX-resistant HT-29 cells. This analysis focused on a large repeat region and complex DNA segments. Finally, the amplified region was characterized and evaluated by size and relevant genetic defects, and potential gene amplification mechanisms were suggested.

To analyze the complicated repetitive sequences involved in gene amplification in the presence of MTX, several MTX-resistant clones were generated from an HT-29 cell line and selected by increasing the MTX concentration in an MTX sensitization procedure. When *DHFR* gene amplification was detected in several clones *via* FISH, the cells in each clone had a very large variation in the 5q12 region. *DHFR* was amplified in a small portion of cells, given that tumor cells responded heterogeneously to MTX. Thus, the effects of the drug can be dissimilar, even when cells are grown under the same conditions^56,57^. This could be associated with bioinformatics difficulties in accurately defining amplification patterns and repetitive sequences, in both previous and future studies.

To overcome the analysis limitations posed by heterogeneous amplification patterns*, DHFR* amplification patterns were analyzed in specific, optimized clones that contained 96% *DHFR*-amplified cells. Approximately 75% of the cells in the clone converged to a homogeneous *DHFR* amplification pattern. After clone optimization, the amplified unit and tandem gene amplification of 11 genes, from the *DHFR* gene to the *ATP6AP1L* gene on chr5 (2.2 Mbp), were characterized on the basis of long-range genomic information obtained from long-read sequencing and optical genome mapping. The amplified unit had high coverage (~197X) compared to the control (~10X). This implied that the amplified region was tandemly amplified by ~20-fold compared to the original sequence, which was confirmed by gene expression analysis demonstrating high levels of transcripts of these genes and their splice variants. Genes within the amplified unit were highly overexpressed, exhibiting 5-fold to 122-fold increases in expression compared to that of the control. The variability of these expression patterns could reflect the complexity of splicing patterns, even if these genes were amplified simultaneously.

Furthermore, inversions at both the start and endpoints of the amplified unit were detected in long-read sequencing data, and this structural variant was cross-validated *via* optical genome mapping. Previous studies have also shown that inversions can lead to the initiation of gene amplification by stimulating chromosomal breakage. In addition, such a phenomenon could represent a transformation from circular DNA segment DMs to intrachromosomal HSRs^58,59^.

Using Hi-C analysis, we identified high levels of intrachromosomal interactions within the amplified region and significant interactions at the novel TADs. An increase in long-range chromosomal interactions from 109 to 138 Mbp, compared with the amplified region, was also unexpectedly detected. The amplified unit in the Hi-C analysis exactly matched that identified by the long-range genomic information and RNA sequencing results. This could indicate that the amplified position was compactly packed such that the amplified sequences had frequent contact with each other, which could reflect the chromatin architecture involved in gene amplification^60^.

In addition, novel frameshift insertions in *MSH* and *MLH* were identified in the MTX-resistant sample, which could play an important role in the rapid progression of gene amplification and MTX resistance. In this vein, microsatellite instability (MSI), i.e., a marker of deficient DNA mismatch repair in colon cancer, was tested to evaluate the status of *MMR* in MTX-resistant HT-29 cells. However, there was no significant difference in MSI between MTX-resistant cells and the control^61^.

Taken together, these findings suggest that *DHFR* is likely not the only target of the MTX-associated mechanism, since 11 previously unimplicated genes were tandemly amplified between *DHFR* and *ATP6AP1L*, and the expanded region on the 5q arm over the amplified region interacted with other amplified genes. The gene in the amplified unit with the highest expression was *RASGRF2*, which exhibited a 122-fold increase compared with the control. Conversely, *DHFR* exhibited only a fivefold increase compared with the control. Therefore, more investigations are needed to target and assess the role of *RASGRF2* in MTX-related gene amplification. This gene might utilize the *DHFR* promoter to achieve its high expression in the presence of MTX.

This implied that MSI did not affect genetic instability and the entire MMR system over whole chromosomes, whereas MTX toxicity could induce mutations in *MMR* genes as well as genetic predisposition to amplification on chromosome 5 in MTX-resistant HT-29 cells through the insertion of adenine or thymine nucleotides into *MSH* and *MLH* genes. This points to a possible tandem gene amplification mechanism that progresses through BFB cycles. In addition, it was possible that BFB cycles under low-level MTX drug selection might generate ecDNAs and chromothripsis, as previously reported^62^. However, we estimate that this is less likely, since no DMs were detected by either FISH or HiC-trans.

Further studies are needed to identify whether a frameshift insertion in *MSH3* might be caused by coamplification of *DHFR* and other frameshift insertions that result in the malfunctioning of mutS homologs and hypermutability in the presence of significant MTX^63,64^. However, frameshift insertions in *MSH* and *MLH* could not be generated by the gene amplification process, since all of the mutated genes, except for *MSH3*, are located outside of the amplified unit. Therefore, the frameshift mutations in each gene should have had a different cause in the presence of MTX toxicity as well as different impacts on MTX-resistant HT-29 cells.

In addition, the mismatch location is recognized by two mutS homologs, mutS-alpha (*MSH2* and *MSH6*), which is known to facilitate the repair of single-nucleotide mismatches, and mutS-beta *(MSH2* and *MSH3*), which is known to assist in the repair of large indels^65,66^. Such mutS homologs should require mutL homologs (*MLH1* and *PMS2*) for binding to a recognition site. Therefore, mutations in mutS and mutL homologs could prevent the repair of single-nucleotide mismatches and large indels in MTX-resistant HT-29 cells. The prevention of mutations and inversions in mutS and mutL homologs could increase cell sensitivity to MTX and inhibit gene amplification.

Although this study has several limitations, our findings may provide new insight into the mechanisms underlying the amplification process and evolution of MTX resistance in colon cancer and leukemia. Moreover, the use of Hi-C data to detect unforeseen chromosomal rearrangements such as DMs and HSRs has been shown to be promising for future analyses.

Most importantly, additional validation of the identified SVs and repetitive sequences is required to interpret the entire sequence of events involved in the gene amplification mechanism and the impact of amplification on MTX resistance. This information will provide clues to understanding how cancers adapt to drugs. Finally, our findings will bolster clinical cancer studies and inform diagnoses as well as the management and treatment of various cancers and provide in-depth guidance toward pharmacologic targets for anticancer drugs as well as personalized medicine.

## Supplementary information

Supplementary Information

Supplementary Data 1

Supplementary Data 2

Supplementary Data 3

Supplementary Data 4

Supplementary Data 5

## Data Availability

The datasets generated and/or analyzed in the current study are available from the corresponding author on reasonable request. Supplementary information accompanies the manuscript on the Experimental & Molecular Medicine website (http://www.nature.com/emm/). The preprint is available on BioRxiv (https://www.biorxiv.org/content/10.1101/2020.02.26.965814v1.abstract)^67^.
