# Supplementary material for: Genomic and transcriptomic analyses reveal a tandem amplification unit of 11 genes and mutations in mismatch repair genes in methotrexate-resistant HT-29 cells
Source: Exp Mol Med. 2021 Sep 14;53(9):1344–55. doi: 10.1038/s12276-021-00668-x (PMC8492700; doi:10.1038/s12276-021-00668-x)
Supplement: Supplementary file 1 — Supplementary Information [file 12276_2021_668_MOESM1_ESM.pdf]

Supplementary Figures 1-15
Supplementary Tables 1-16
Supplementary Data 1-5

Supplementary Figures List

Supplementary Figure 1. Schematic workflow.
Supplementary Figure 2. The morphological change of MTX resistant colon cancer cells.
Supplementary Figure 3. The detection of the amplified *DHFR* gene at 5q arm in MTX resistant C1-2.
Supplementary Figure 4. The visualization of the amplified *DHFR* gene at 5q arm in MTX resistant C1-2.
Supplementary Figure 5. The detected FISH patterns of amplified *DHFR* gene in MTX resistant C1-2.
Supplementary Figure 6. The visualization of inter-chromosomal genomic rearrangements in MTX resistant HT-29 and control samples.
Supplementary Figure 7. The multiple-read view of alignment over amplified region with Ribbon.
Supplementary Figure 8. The comparison of Bionano contigs and PacBio PBcR contigs over amplified region
Supplementary Figure 9. The scaffolding of PacBio long reads over amplified region compared to hg 38.
Supplementary Figure 10. Genome mapping over the amplified region.
Supplementary Figure 11. The expression level in 5q 14.2 region.
Supplementary Figure 12. The difference of non-synonymous mutations between MTX resistant HT-29 and control over whole chromosomes.
Supplementary Figure 13. Genome-wide view of intra-chromosomal interactions.
Supplementary Figure 14. The topologically associating domains (TADs) on chromosome 5.
Supplementary Figure 15. The comparison of relative copy number between MTX resistant and control samples.

Supplementary Tables List

Supplementary Table 1. RNA mapping statistics
Supplementary Table 2. PacBio mapping statistics
Supplementary Table 3. The summary of PBcR assembly over amplified region
Supplementary Table 4. The summary of Bionano contigs and PacBio PBcR contigs over amplified region.
Supplementary Table 5. Bionano raw data statistics
Supplementary Table 6. Bionano assembly statistics
Supplementary Table 7. Hi-C mapping statistics
Supplementary Table 8. The estimation of copy number and expression of *DHFR* gene in MTX resistant clone (C1-2) at each cycle.
Supplementary Table 9. The detected variants on chromosome 5 in MTX resistant HT-29
Supplementary Table 10. The comparison of alternative splicing patterns on the duplication unit between MTX resistant and control samples.
Supplementary Table 11. The comparison of mutations between control and MTX resistant HT-29 over whole chromosomes.
Supplementary Table 12. The comparison of mutations between control and MTX resistant HT-29 on chromosome 5.
Supplementary Table 13. The detection of novel frameshift insertions in MTX resistant HT-29 compared to control.
Supplementary Table 14. The differentially expressed genes (DEGs) in MTX resistant HT-29.
Supplementary Table 15. The topologically associating domains (TADs) with high intra-chromosomal interactions on chromosome 5.
Supplementary Table 16. The transportable elements of gene amplified region in MTX-resistant sample

Supplementary Data List

Supplementary Data 1. The filtered variants and CNV category in control
Supplementary Data 2. The filtered variants and CNV category in MTX resistant HT-29
Supplementary Data 3. The comparison of detected somatic copy number aberrations between control and MTX resistant HT-29
Supplementary Data 4. The detected mutations in MTX resistant HT-29
Supplementary Data 5. The detected mutations in control

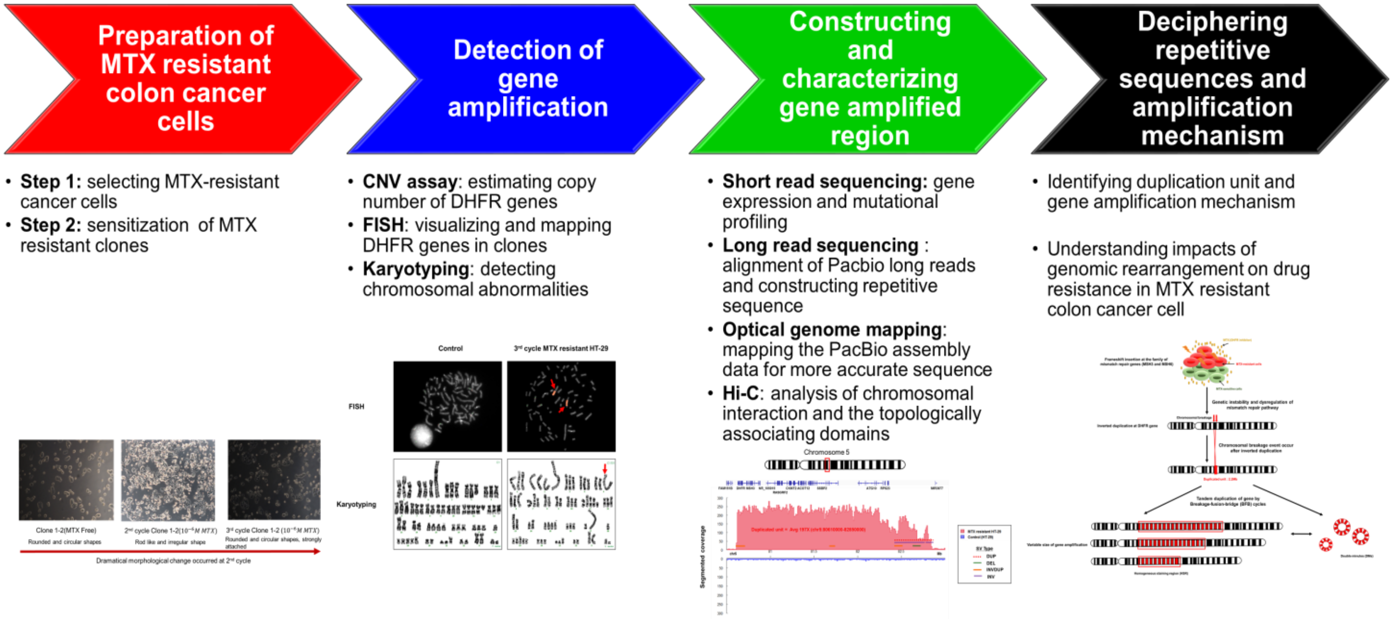

Supplementary Figure 1. Overview of the study workflow. The experimental and computational analyses were performed on methotrexate resistant colon cancer cell line (HT-29) in order to characterize gene amplified region and understand the underlying mechanism.

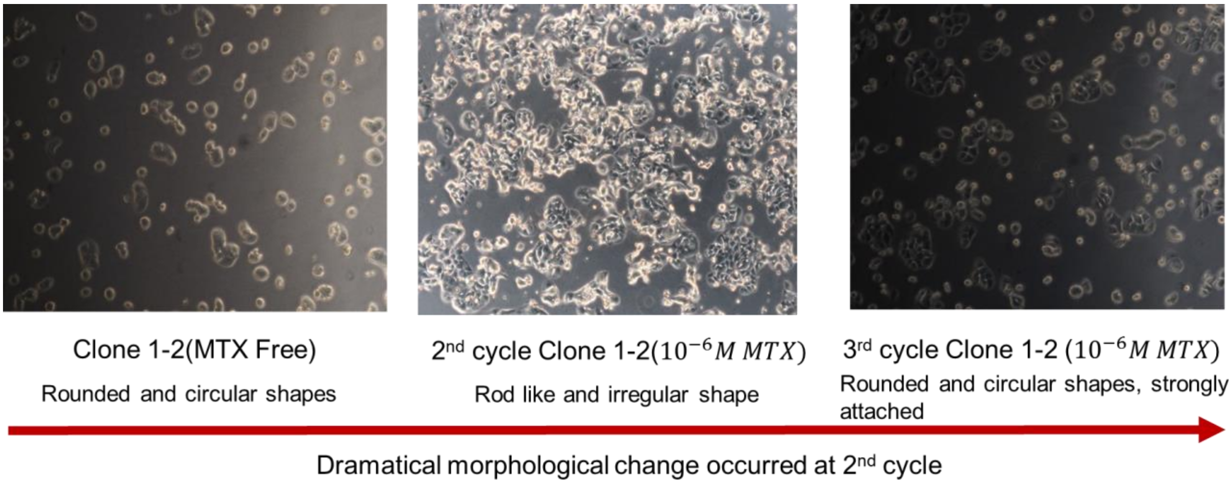

Supplementary Figure 2. The morphological change of MTX resistant colon cancer cells. The MTX resistant clone (C1-2) was detected and captured by light microscope at 200x magnification. The morphological changes at each cycle were indicated under 10^{-6} mol/L MTX.

a

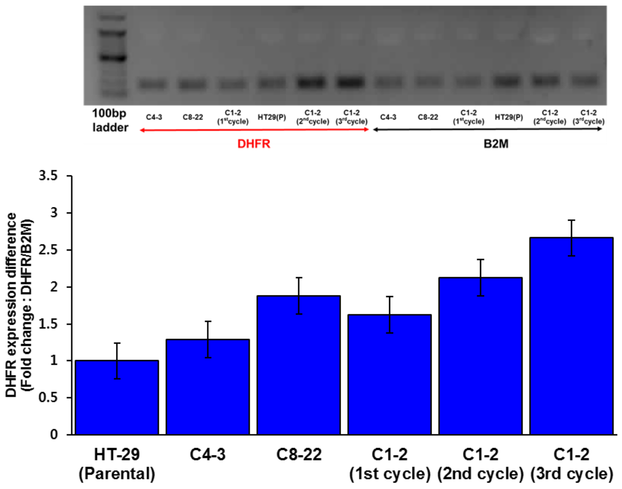

b

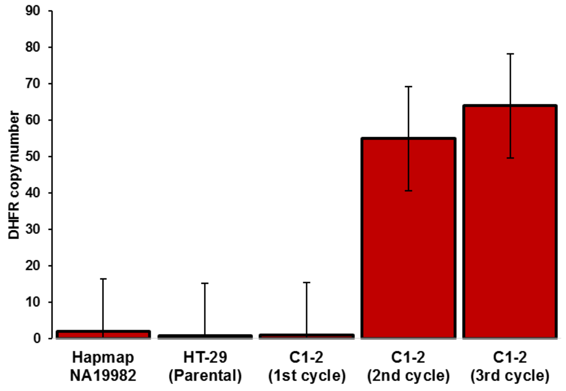

Supplementary Figure 3. The detection of the amplified *DHFR* gene at 5q arm in MTX resistant C1-2. The expression (a) and copy number (b) of *DHFR* gene in MTX resistant clones and C1-2 clone at each cycle were estimated by qPCR. The error bars indicated standard error (SE) of the mean.

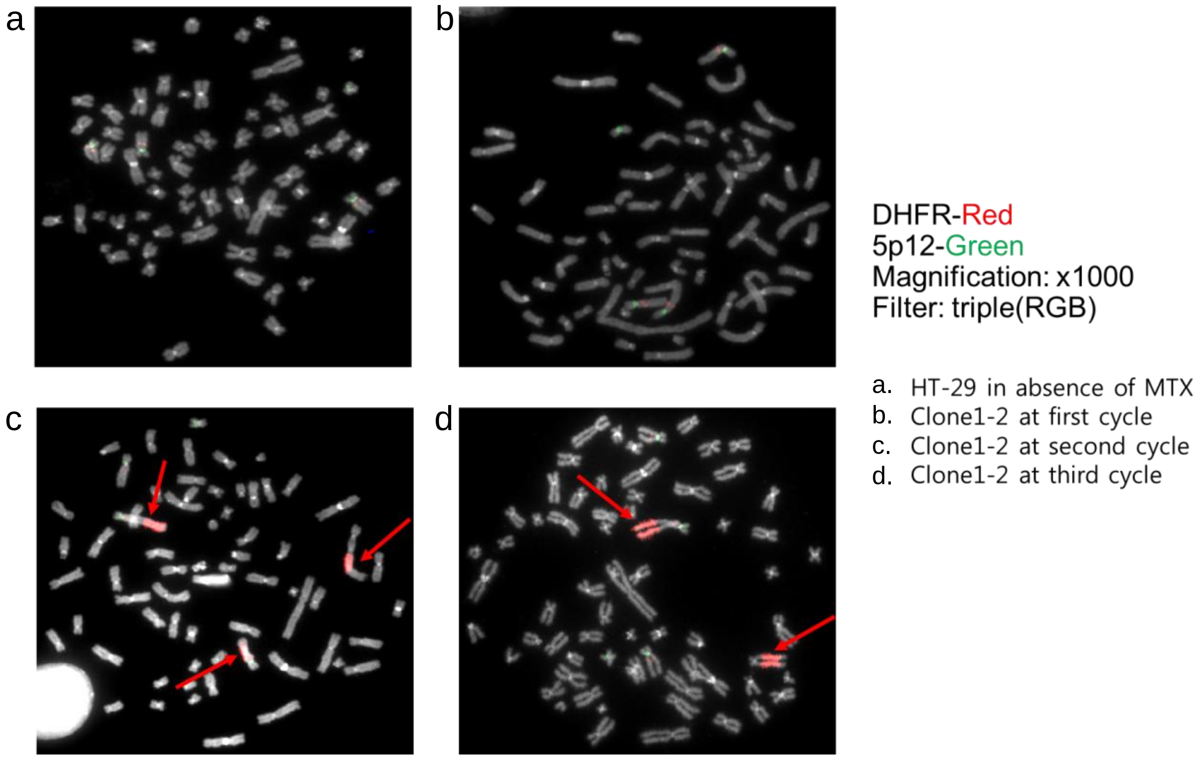

Supplementary Figure 4. The visualization of the amplified *DHFR* gene at 5q arm in MTX resistant C1-2. The amplified DHFR gene was visualized by Fluorescence In Situ Hybridization (FISH) on MTX resistant clone (C1-2) at each cycle and control sample with the 1000x magnification. The amplified *DHFR* gene region was indicated by the red arrow.

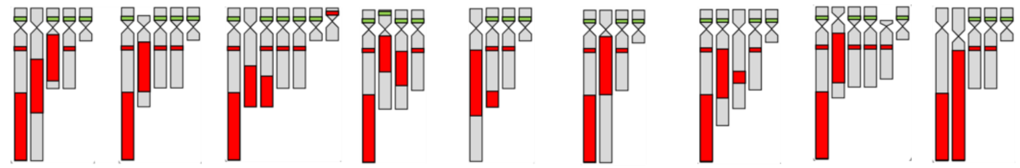

	Type 1	Type 2	Type 3	Type 4	Type 5	Type 6	Type 7	Type 8	Type 9
**DHFR-Red Signal count**	P3,S2	P2,S3	P3,S5	P3,S2	P2,S2	P2,S2	P3,S2	P2,S4	P2,S2
**5p12-Green signal count**	4	4	5	4	3	3	4	4	3
**Metaphase count (%)**	11 (44)	7 (28)	1 (4)	1 (4)	1 (4)	1 (4)	1 (4)	1 (4)	1 (4)

Supplementary Figure 5. The detected FISH patterns of amplified *DHFR* gene in MTX resistant C1-2. The FISH signal type of amplified DHFR gene in MTX resistant clone (C1-2) was displayed with the paining signal (P) and spot signal (S). 5q12 was indicated by green signal, and *DHFR* gene was indicated by red signal. Each number of red and green signal and metaphase were counted.

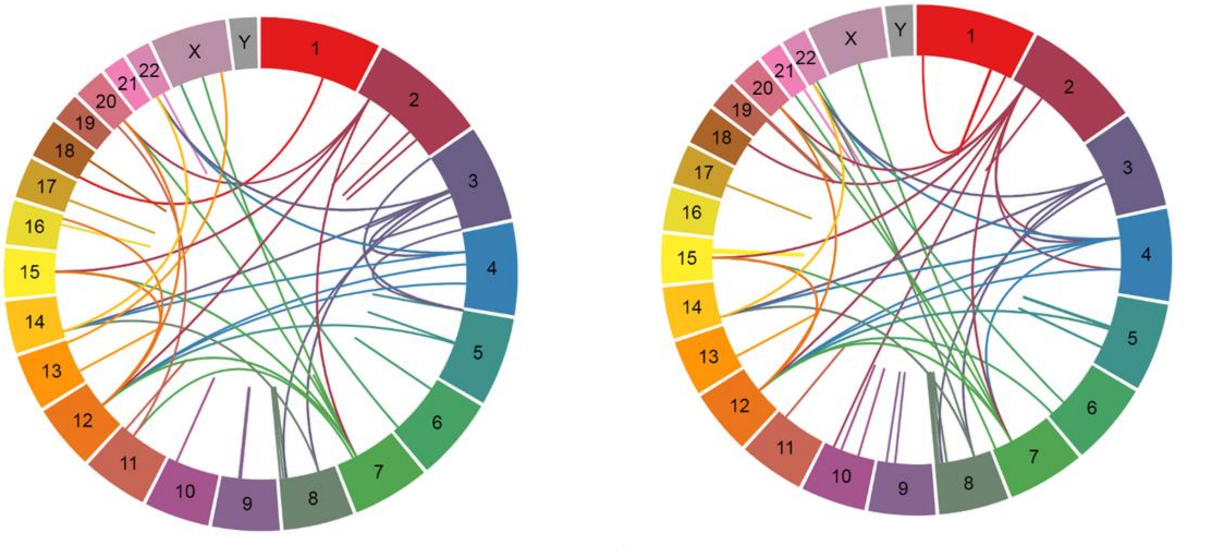

Control (HT29)   MTX resistant HT29

Supplementary Figure 6. The visualization of inter-chromosomal genomic rearrangements in MTX resistant HT-29 and control samples. The inter-chromosomal genomic rearrangements were compared between MTX resistant HT-29 and control samples, and it was visualized by Splitthreader over all chromosomes.

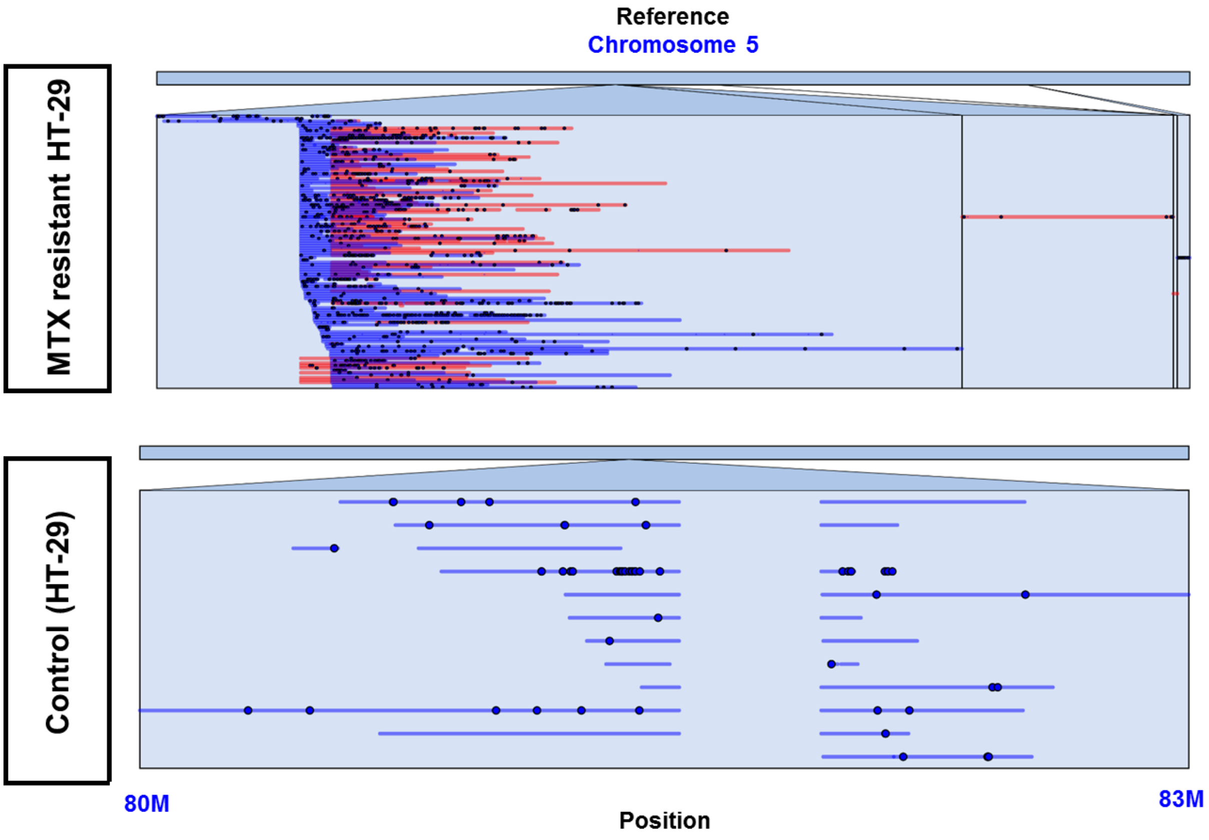

Supplementary Figure 7. The multiple-read view of alignment over amplified region with Ribbon. The multi-reads from alignment data (BAM) was visualized and compared between control and MTX resistant sample on chromosome 5: 80,000,000-83,000,000 by using Ribbon. Blue and red lines indicated the forward and reverse strands, respectively.

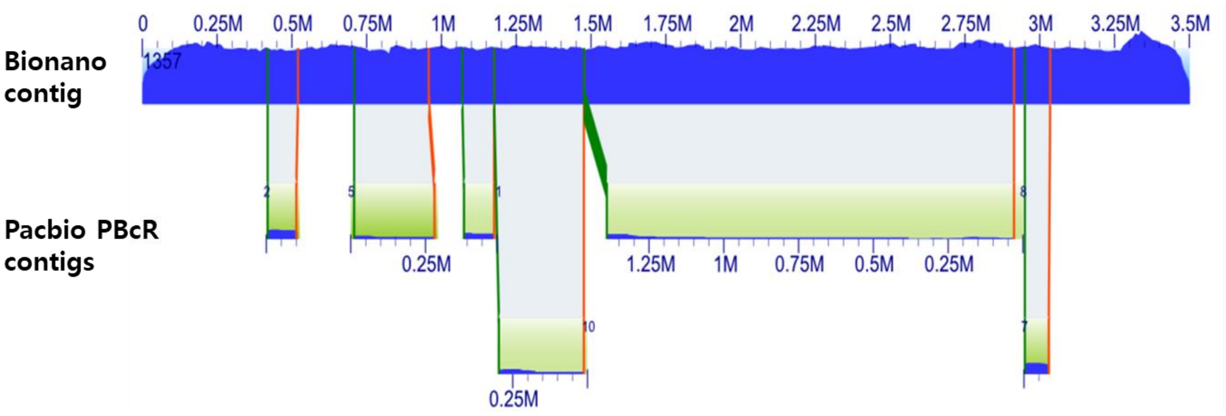

Supplementary Figure 8. The comparison of Bionano contigs and PacBio PBcR contigs over amplified region. The PacBio assembled contigs matched up with BioNano contigs on amplified region, and the start and end position of contigs were indicated by green and orange lines, respectively.

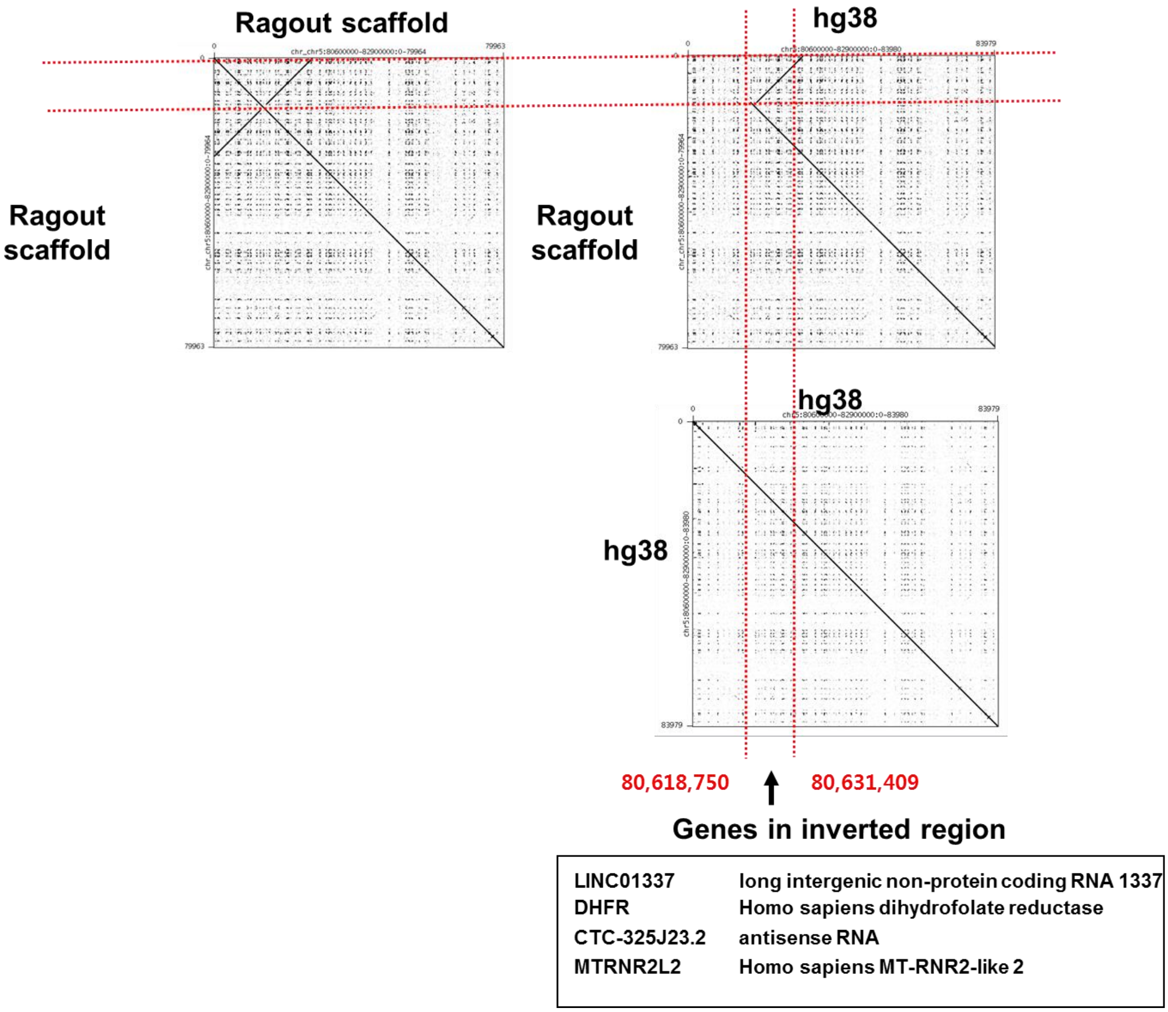

Supplementary Figure 9. The scaffolding of PacBio long reads over amplified region compared to hg 38. The inversion at the start point of amplification unit was visualized by the three dot plots compared to hg38.The inverted region and its genes were indicated with the description.

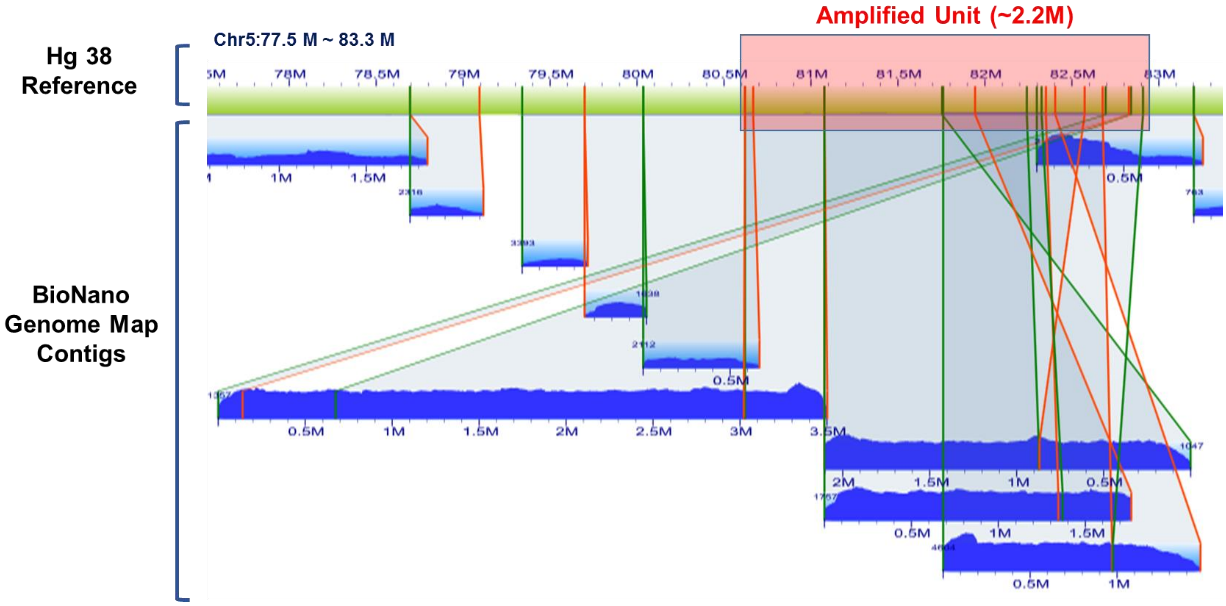

Supplementary Figure 10. Genome mapping over the amplified region. The BioNano genome map contigs around amplified region were visualized and compared along with the reference (hg38). The start position of contig indicated with the green color, and the end position of contig indicated with the orange color.

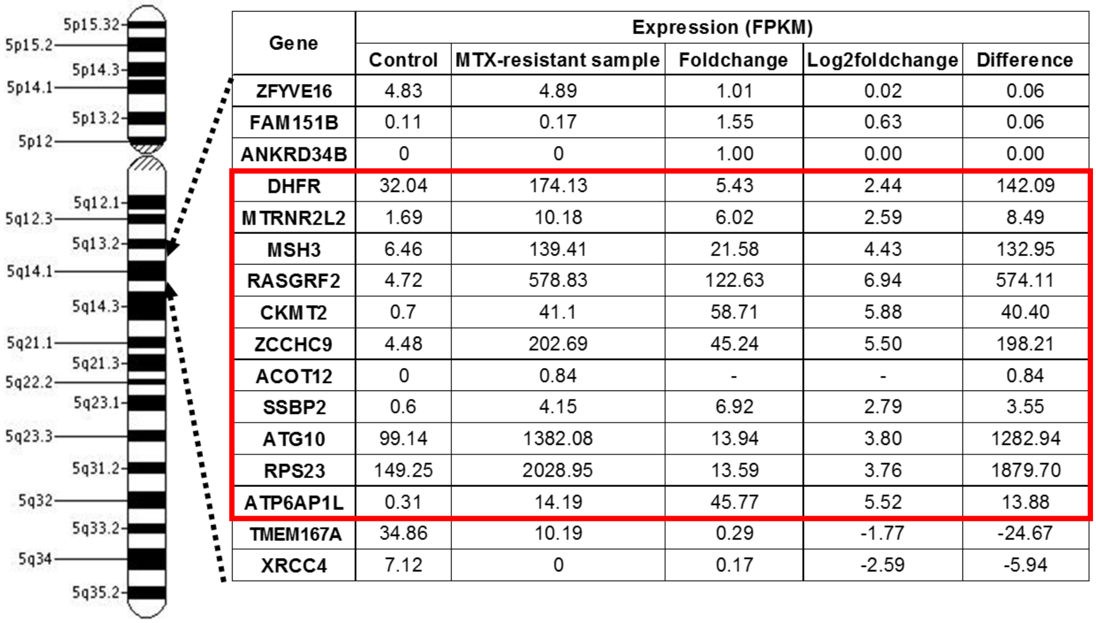

Gene	Expression (FPKM)				
	Control	MTX-resistant sample	Foldchange	Log2foldchange	Difference
ZFYVE16	4.83	4.89	1.01	0.02	0.06
FAM151B	0.11	0.17	1.55	0.63	0.06
ANKRD34B	0	0	1.00	0.00	0.00
DHFR	32.04	174.13	5.43	2.44	142.09
MTRNR2L2	1.69	10.18	6.02	2.59	8.49
MSH3	6.46	139.41	21.58	4.43	132.95
RASGRF2	4.72	578.83	122.63	6.94	574.11
CKMT2	0.7	41.1	58.71	5.88	40.40
ZCCHC9	4.48	202.69	45.24	5.50	198.21
ACOT12	0	0.84	-	-	0.84
SSBP2	0.6	4.15	6.92	2.79	3.55
ATG10	99.14	1382.08	13.94	3.80	1282.94
RPS23	149.25	2028.95	13.59	3.76	1879.70
ATP6AP1L	0.31	14.19	45.77	5.52	13.88
TMEM167A	34.86	10.19	0.29	-1.77	-24.67
XRCC4	7.12	0	0.17	-2.59	-5.94

Supplementary Figure 11. The expression level in 5q 14.2 region. The table depicted the expression level (FPKM) over duplicated region, and the fold change and difference of expression were computed between control and MTX resistant HT-29. The fold change, which is bigger than 5, was indicated with the red box. .

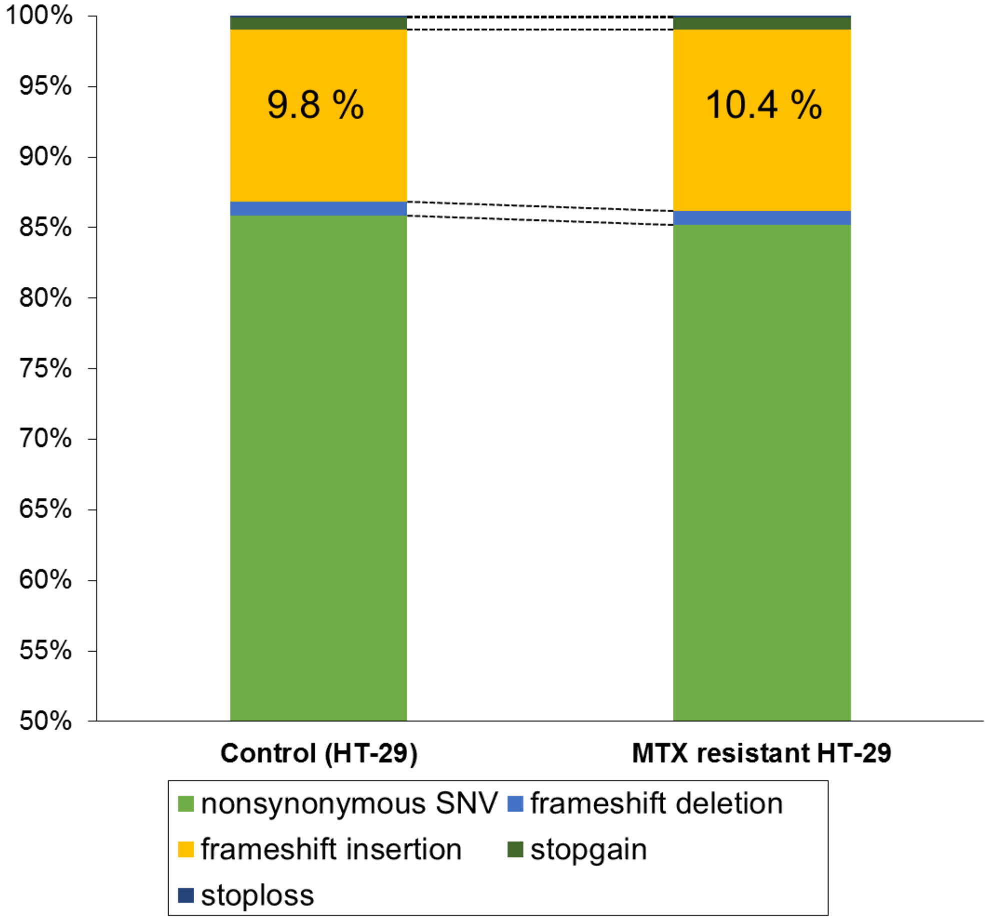

Supplementary Figure 12. The difference of non-synonymous mutations between MTX resistant HT-29 and control over whole chromosomes. The non-synonymous mutations (non-synonymous SNV, frameshift deletion, frameshift insertion, stop-gain, and stop-loss) were compared between MTX resistant HT-29 and control over whole chromosomes, and the percentage of frameshift insertions was indicated.

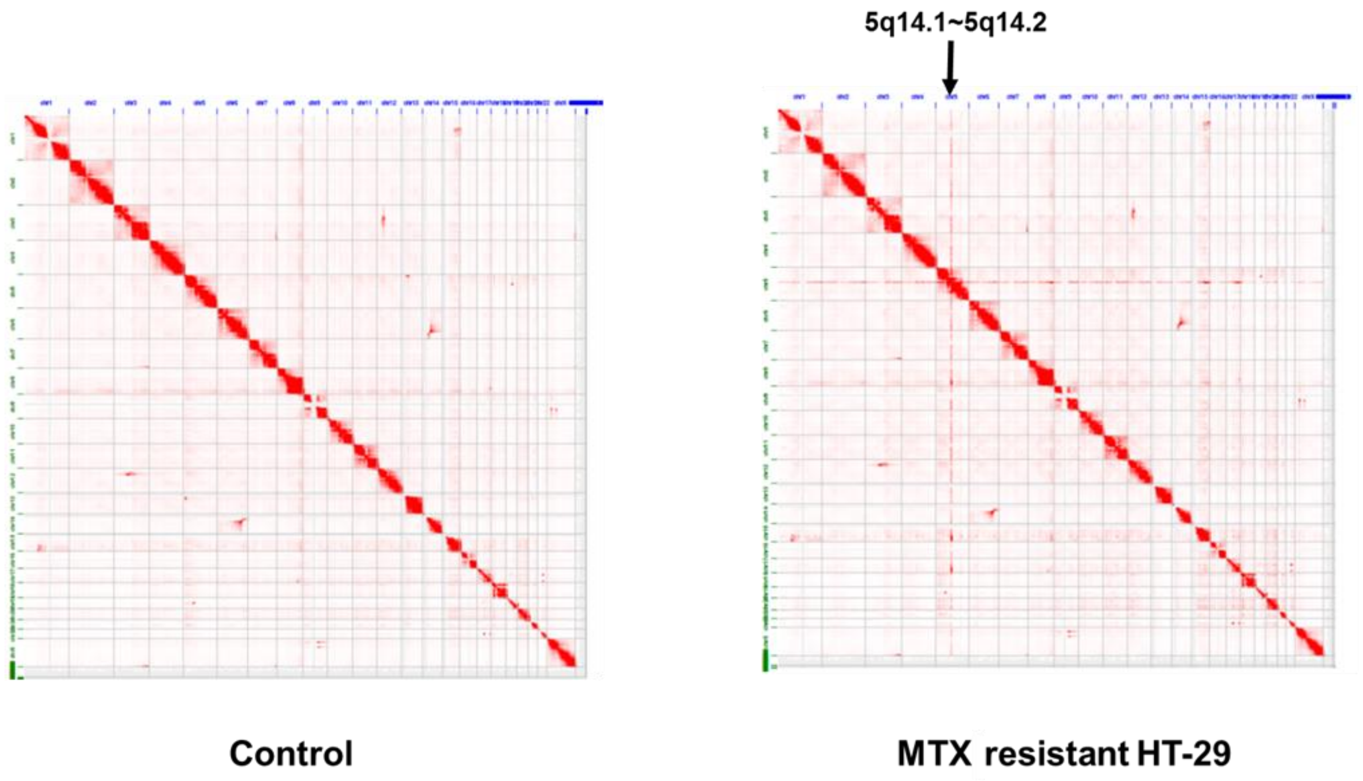

Supplementary Figure 13. Genome-wide view of intra-chromosomal interactions. The intra-chromosomal interactions over genome-wide view in MTX resistant HT-29 and control were visualized by Juicebox at 5kb resolution, and the amplified region was indicated by the arrow.

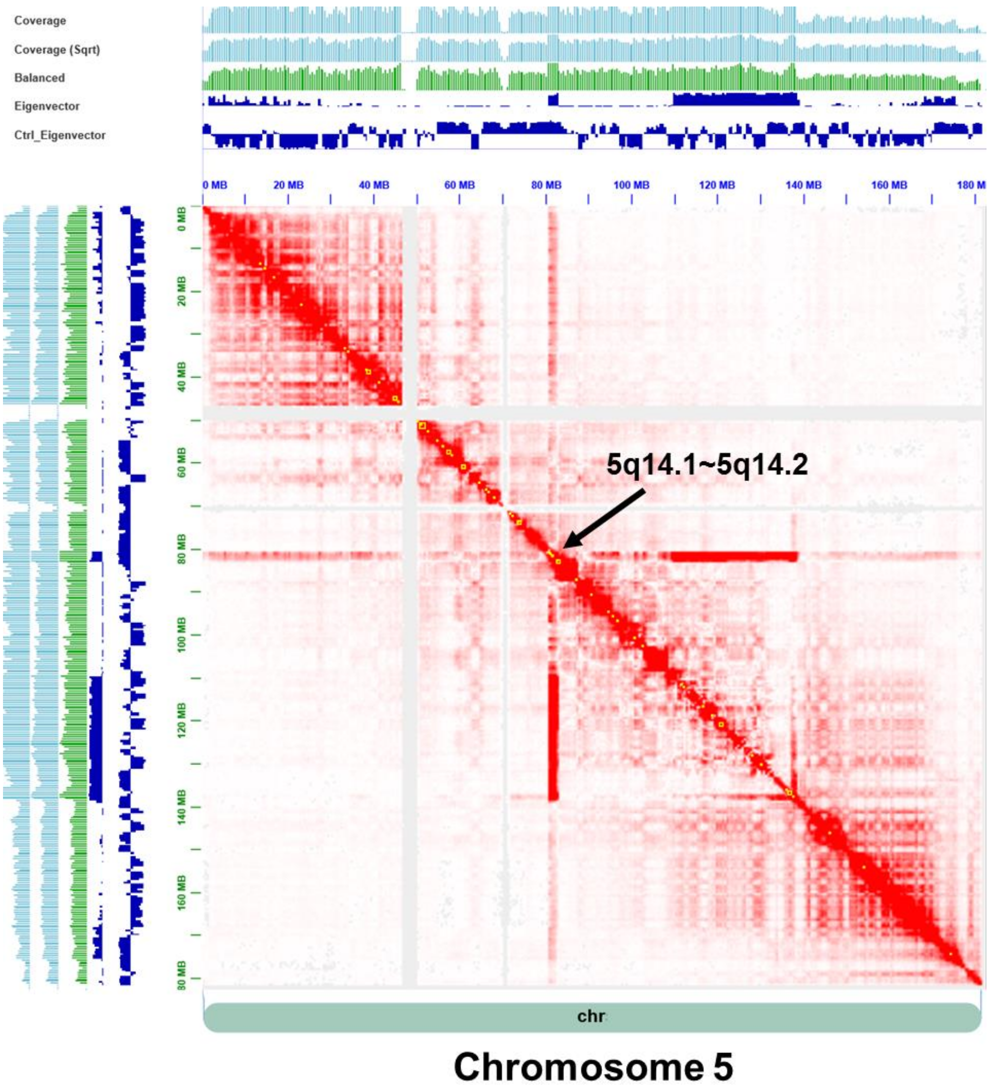

Supplementary Figure 14. The topologically associating domains (TADs) on chromosome 5. The topologically associating domains were identified by Arrowhead algorisms and visualized with the intra-chromosomal interactions on chromosome 5. The TADs were yellow-boxed.

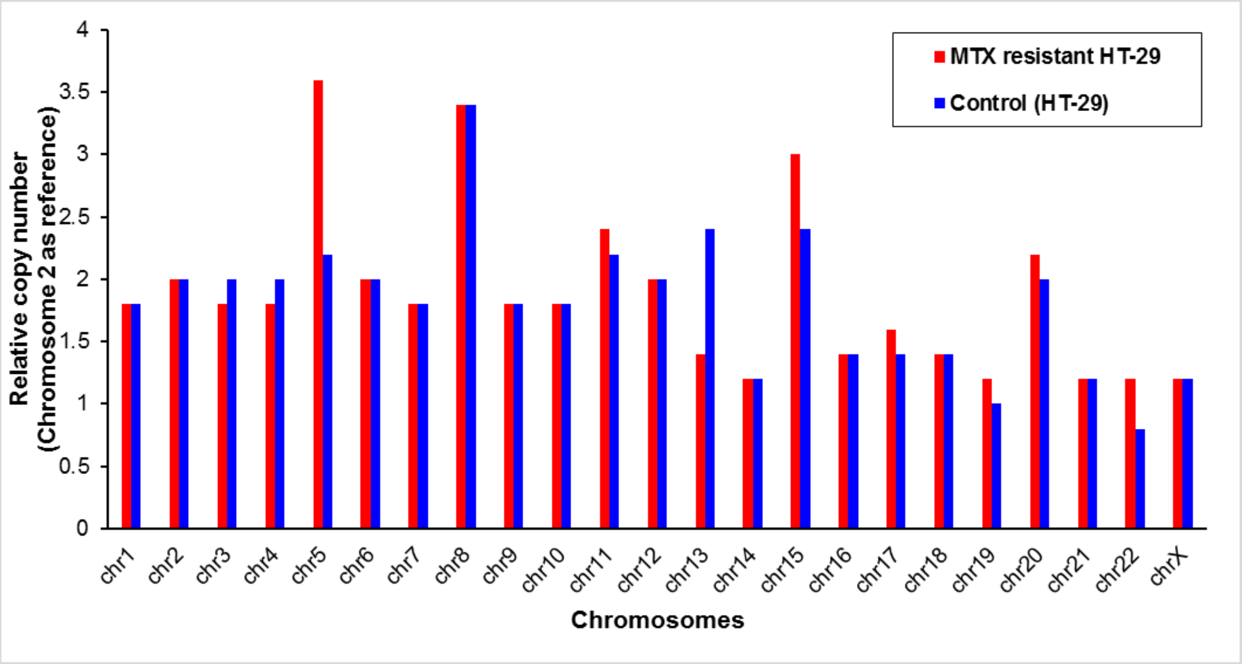

Supplementary Figure 15. The comparison of relative copy number between MTX resistant and control samples. The relative copy number over whole chromosomes was estimated by HiCnv from Hi-C data. The copy number of chromosome 2 was used as the reference.

Supplementary Table 1. RNA mapping statistics

Statistics	Control	MTX resistant HT-[illegible]
**Number of input reads**	130504160	112834920
**Average input read length**	202	202
**UNIQUE READS**		
**Uniquely mapped reads number**	124006754	104802461
**Uniquely mapped reads %**	95.02%	92.88%
**Average mapped length**	200.81	200.68
**Number of splices: Total**	91567846	38317556
**Number of splices: Annotated (sjdb)**	90856209	37668649
**Number of splices: GT/AG**	90763207	37973986
**Number of splices: GC/AG**	715982	316984
**Number of splices: AT/AC**	88657	26586
**Number of splices: Non-canonical**	0	0
**Mismatch rate per base, %**	0.35%	0.39%
**Deletion rate per base**	0.01%	0.01%
**Deletion average length**	1.66	1.61
**Insertion rate per base**	0.01%	0.01%
**Insertion average length**	1.39	1.3
**MULTI-MAPPING READS**		
**Number of reads mapped to multiple loci**	4051446	4533586
**% of reads mapped to multiple loci**	3.10%	4.02%
**Number of reads mapped to too many loci**	37487	116342
**% of reads mapped to too many loci**	0.03%	0.10%
**UNMAPPED READS**		
**% of reads unmapped: too many mismatches**	0.00%	0.00%
**% of reads unmapped: too short**	1.83%	2.96%
**% of reads unmapped: other**	0.02%	0.04%

Supplementary Table 2. PacBio mapping statistics

Statistics	Control	MTX resistant HT-29
**Average mapping rate**	0.551328	0.568679
**QC-passed reads**	2307856	2986204
**Supplementary reads**	692063	1006884
**Mapped reads**	2260872(97.96%)	2922821(97.88%)
**Average aligned region depth**	4.85289	5.38979
**Paired in sequencing (total reads)**	3231586	3958640
**With itself and mate mapped**	3137618	3831874

Supplementary Table 3. The summary of PBcR assembly over amplified region

Assembly	#Contigs	Contigs sum	N50	Longest	Shortest	Average
**PBcR contigs**	10	2,382,509	1,391,177	1,391,177	18,181	238,250
**Scaffold using Ragout**	1	2,297,321	2,297,321	2,297,321	2,297,321	2,297,321

Supplementary Table 4. The summary of Bionano contigs and PacBio PBcR contigs over amplified region.

Anchor_ID	AnchorStart	AnchorEnd	align size	Qry_ID	ctg	ctg size	Qry_Start	Qry_End	Orient	Confindence
1357	418,977	520,390	101,413	2	ctg71800 00000011	111,775	4,355	100,463	+	12.01
1357	708,068	957,449	249,382	5	ctg71800 00000014	291,638	11,366	280,084	+	35.87
1357	1,069,628	1,176,192	106,564	1	ctg71800 00000010	114,900	9,497	110,367	-	16.7
1357	1,176,192	1,476,135	299,943	10	ctg71800 00000019	298,862	12,163	296,385	-	35.73
1357	1,476,135	2,914,027	1,437,892	8	ctg71800 00000017	1,391,177	29,838	1,390,934	-	168.4
1357	2,950,166	3,034,883	84,717	7	ctg71800 00000016	87,555	2,838	82,950	+	13.06

Supplementary Table 5. Bionano raw data statistics

Statistic	Control	MTX resistant HT-29
**Total DNA (Kb)**	207,254,999	249,234,905
**Total Molecules (>100kb)**	810,590	1,070,988
**Occupancy**	4.56%	5.48%
**Average Molecule SNR**	10.05	9.47
**Average Molecule Intensity**	0.25	0.4
**Center of Mass**	219.49	260.46
**Average Length (Kb)**	255.68	232.71
**Avgerage Label Density (per 100kb)**	8.4	8.52

Supplementary Table 6. Bionano assembly statistics

Statistic	Control	MTX resistant HT-29
**Mapped Molecule Quantity (Mb)**	128,063.90	136,651.81
**Mapped Average Size (Kb)**	319	304
**Average Label Density (per 100 Kb)**	8.8	8.9
**Number of Consensus Genome Maps**	3046	3474
**Consensus Genome Maps Size (Mb)**	2849.828	2850.691
**Consensus Genome Maps N50 (Mb)**	1.262	1.064
**Average Depth of Mol Coverage**	33.4	34.6

Supplementary Table 7. Hi-C mapping statistics

Statistic	Control	MTX resistant HT-29
**Sequenced Read Pairs**	946,011,579	783,851,679
**Normal paired**	629,287,307 (66.52%)	523,590,997 (66.80%)
**Chimeric Paired**	259,322,968 (27.41%)	213,102,828 (27.19%)
**Chimeric Ambiguous**	48,168,247 (5.09%)	39,030,865 (4.98%)
**Unmapped**	9,233,067 (0.98%)	8,126,989 (1.04%)
**Ligation Motif Present**	591,982,478 (62.58%)	481,312,778 (61.40%)
**Alignable (Normal+Chimeric Paired)**	888,610,265 (93.93%)	736,693,825 (93.98%)
**Intra-fragment Reads**	30,657,735 (3.24% / 3.96%)	33,601,960 (4.29% / 5.12%)
**Below MAPQ Threshold**	98,430,220 (10.40% / 12.70%)	81,523,351 (10.40% / 12.42%)
**Hi-C Contacts**	645,867,029 (68.27% / 83.34 %)	541,469,462 (69.08% / 82.47 %)
**Ligation Motif Present**	304,551,801 (32.19% / 39.30%)	254,351,744 (32.45% / 38.74%)
**3' Bias (Long Range)**	94% - 6%	94% - 6%
**Pair Type %(L-I-O-R)**	25% - 25% - 25% - 25%	25% - 25% - 25% - 25%
**Inter-chromosomal:**	74,505,389 (7.88% / 9.61%)	61,489,287 (7.84% / 9.36%)
**Intra-chromosomal:**	571,361,640 (60.40% / 73.73%)	479,980,175 (61.23% / 73.10%)
**Short Range (<20Kb)**	185,326,880 (19.59% / 23.91%)	155,045,349 (19.78% / 23.61%)
**Long Range (>20Kb)**	386,034,531 (40.81% / 49.81%)	324,934,625 (41.45% / 49.49%)

Supplementary Table 8. The estimation of copy number and expression of *DHFR* gene in MTX resistant clone (C1-2) at each cycle.

Type of Sample	Sample Ct	Expression	Rate for Copy Number	Copy Number
Hapmap NA19982	24.99	2.52	1.00	2.00
Control (HT-29)	26.33	1.00	0.40	0.79
1st cycle MTX resistant HT-29	26.04	1.22	0.48	0.97
2nd cycle MTX resistant HT-29	20.21	69.17	27.42	54.83
3rd cycle MTX resistant HT-29	19.99	80.56	31.93	63.87

Supplementary Table 9. The detected variants on chromosome 5 in MTX resistant HT-29

chrom1	pos1	strand1	chrom2	pos2	strand2	variant_name	variant_type	split	size	CNV_category	category	nearby_variant_count
5	80617089	-	5	80618750	-	24	INVDUP	47	1661	matching	simple	0
5	81781592	+	5	81781648	+	50	INVDUP	10	56	neutral	simple	0
5	82245207	+	5	82573904	+	56	INV	56	328697	matching	crowded	1
5	82470789	-	5	82842464	+	58	DUP	11	371675	matching	crowded	4
5	82470887	+	5	82804501	+	59	INV	12	333614	partial	crowded	4
5	82473031	-	5	82474560	-	60	INVDUP	18	1529	matching	simple	3
5	82678370	+	5	82764579	-	62	DEL	56	86209	matching	simple	3
5	82804502	+	5	82842464	+	64	INV	47	37962	matching	simple	3
5	137519108	+	5	137520622	-	65	DEL	19	1514	neutral	simple	0
5	137686889	+	5	137688197	-	66	DEL	22	1308	neutral	simple	0
5	88112313	-	12	66057592	-	256	TRA	12	-1	neutral	reciprocal	12
5	88112381	+	12	66057683	+	284	TRA	12	-1	neutral	reciprocal	12

Supplementary Table 10. The comparison of alternative splicing patterns on the duplication unit between MTX resistant and control samples.

EventType	SigEvents.JC+readsOnTarget	
	MTX resistant HT-29	Control (HT-29)
**SE**	3086	1732
**MXE**	785	3231
**A5SS**	526	169
**A3SS**	943	228
**RI**	2378	148

Abbreviations
Skipped exon (SE)
Mutually exclusive exon (MXE)
Alternative 5' splice site (A5SS)
Alternative 3' splice site (A3SS)
Retained intron (RI)

Supplementary Table 11. The comparison of mutations between control and MTX resistant HT-29 over whole chromosomes.

Mutation types	MTX resistant HT-29	Control (HT-29)
Exonic total	13982	13310
Exonic splicing	10	9
Splicing	70	155
ncRNA exonic	6040	5876
ncRNA exonic:splicing	2	3
ncRNA splicing	21	26
3'UTR	28523	27892
5'UTR	3448	3632
Nonsynonymous SNV	5708	5730
Frameshift deletion	70	66
Frameshift insertion	858	816
Stopgain	57	55
Stoploss	9	9

Supplementary Table 12. The comparison of mutations between control and MTX resistant HT-29 on chromosome 5.

Mutation types	MTX resistant HT-29	Control (HT-29)
Exonic total	577	559
Exonic splicing	1	0
Splicing	4	8
ncRNA exonic	159	206
ncRNA exonic:splicing	0	0
ncRNA splicing	1	1
3'UTR	1332	1337
5'UTR	166	164
Nonsynonymous SNV	220	226
Frameshift deletion	4	3
Frameshift insertion	51	12
Stopgain	6	4
Stoploss	0	0

Supplementary Table 13. The detection of novel frameshift insertions in MTX resistant HT-29 compared to control.

MTX resistant HT-29									
**Chr**	**Start**	**End**	**Ref**	**Alt**	**Func.refGene**	**Gene.refGene**	**Gene Title**	**ExonicFunc.refGene**	**ExAC_ALL**
chr2	47803552	47803552	-	T	exonic	MSH6	mutS homolog 6	frameshift insertion	.
chr2	189818083	189818083	-	A	exonic	PMS1	postmeiotic segregation increased 1	frameshift insertion	3.54E-05
chr2	189863838	189863838	-	A	exonic	PMS1	postmeiotic segregation increased 1	frameshift insertion	.
chr3	37012077	37012077	A	G	exonic	MLH1	mutL homolog 1	nonsynonymous SNV	0.2325
chr5	80654962	80654962	A	G	exonic	MSH3	mutS homolog 3	nonsynonymous SNV	0.9029
chr5	80675095	80675095	-	A	exonic	MSH3	mutS homolog 3	frameshift insertion	.
chr5	80854162	80854162	A	G	exonic	MSH3	mutS homolog 3	nonsynonymous SNV	0.8731
chr5	80873118	80873118	G	A	exonic	MSH3	mutS homolog 3	nonsynonymous SNV	0.7305
chr7	5987144	5987144	T	C	exonic	PMS2	postmeiotic segregation increased 2	nonsynonymous SNV	0.8514
chr7	5987357	5987357	G	A	exonic	PMS2	postmeiotic segregation increased 2	nonsynonymous SNV	0.3854
chr7	5987525	5987525	-	T	exonic	PMS2	postmeiotic segregation increased 2	frameshift insertion	.
chr14	75047125	75047125	G	A	exonic	MLH3	mutL homolog 3	nonsynonymous SNV	0.4126
chr14	75047180	75047180	T	C	exonic	MLH3	mutL homolog 3	nonsynonymous SNV	0.9968
**Control (HT-29)**									
**Chr**	**Start**	**End**	**Ref**	**Alt**	**Func.refGene**	**Gene.refGene**	**Gene Title**	**ExonicFunc.refGene**	**ExAC_ALL**
chr3	37012077	37012077	A	G	exonic	MLH1	mutL homolog 1	nonsynonymous SNV	0.2325
chr5	80654905	80654905	-	CCGCAGCGC	exonic	MSH3	mutS homolog 3	nonframeshift insertion	0.0427
chr5	80654962	80654962	A	G	exonic	MSH3	mutS homolog 3	nonsynonymous SNV	0.9029
chr5	80854162	80854162	A	G	exonic	MSH3	mutS homolog 3	nonsynonymous SNV	0.8731
chr5	80873118	80873118	G	A	exonic	MSH3	mutS homolog 3	nonsynonymous SNV	0.7305
chr7	5987144	5987144	T	C	exonic	PMS2	postmeiotic segregation increased 2	nonsynonymous SNV	0.8514
chr7	5987357	5987357	G	A	exonic	PMS2	postmeiotic segregation increased 2	nonsynonymous SNV	0.3854
chr14	75047125	75047125	G	A	exonic	MLH3	mutL homolog 3	nonsynonymous SNV	0.4126
chr14	75047180	75047180	T	C	exonic	MLH3	mutL homolog 3	nonsynonymous SNV	0.9968

Supplementary Table 14. The differentially expressed genes (DEGs) in MTX resistant HT-29.

Up-regulated DEGs (383 genes)	PRSS22 BAIAP3 ETV7 CD22 ALOX5 TYMP BTN3A1 USP2 TRAF1 SLC2A3 CACNB1 GPR116 CLEC2D TRIB2 SPEG SIDT1 GSDMB DHRS9 SCARF1 CACNG4 RGS11 P2RX5 COL16A1 HSD17B14 ASAP3 EPB41L1 TMEM40 SLC4A11 LAG3 ARHGAP4 ANKRD24 YPEL3 PITPNM2 MYO15A NLRP1 DFNB31 CATSPERG CYP2D6 APOL4 UPK3A REC8 WFDC2 MAP1LC3A ATP11C KCND1 VGLL1 GABRE GDPD3 BMF RHOV MAP4K1 AMH IL4I1 DENND3 CCDC114 PBX4 SH3GL2 UNC5B MAP3K8 RASD1 DHX58 MYH3 MGP ADTRP ULBP1 MSH3 RASGRF2 HES1 SLC4A3 PCSK4 FN1 MLPH PADI2 GBP1 SGK1 CTGF TRPM6 LTBP2 GPR68 DUSP1 CD274 PLEKHG1 TP53AIP1 EGR1 TNFSF10 EGR2 NR4A1 C4BPB ARHGAP40 PMEPA1 ZBP1 PRICKLE4 CDKN1A RUNX2 C20orf195 IL1B C3 FOSB ID1 PRKCG ZFP36 APOL3 APOL2 APOBEC3F KRT17 LOXL1 CHRNA10 APOE TNNI2 PNPLA7 ANGPTL6 C19orf66 PLXNA3 SH3BP5 PDLIM4 CKMT2 ZCCHC9 LGALS3 TRIM22 XAF1 REEP2 ALDH3B2 FBXO44 EPSTI1 RARRES3 RSAD2 SLC37A2 ANXA1 ELF5 EGR4 WNT10A THSD1 SCEL RTP4 CASP1 IFI44L MYOF LOXL4 DUSP5 GPR87 ADAMTS14 SEMA7A RASGEF1B ITGB7 ITGAX ZMYND15 ATHL1 SIK1 CACNG8 PADI1 SYTL1 CYR61 CTSK REN CSRNP1 KLHL3 SERPING1 LYPD6B IL18 ATG10 ACOXL NR4A2 GPR110 ANKRD29 CERS3 GOLGA7B UBE2L6 TNFRSF14 DUSP2 XDH BTG2 CCDC17 PAQR6 FOXH1 SLC25A34 SCNN1D GBP4 ATF3 IVL ELF3 IFI16 KIAA1407 ZC3H12A SHROOM1 TRPV6 OTUD1 DKFZP686J19100 HDGFRP3 ANKDD1A CERCAM PRRT2 KIFC2 RILP TRPV3 TMEM88 TRANK1 TTC21A MFSD7 THBS3 CXCL10 IL8 TMEM154 FOS CST1 STAT2 CEL KRT13 EFEMP2 SYT12 C11orf80 TDRD12 SLC26A9 SLC22A1 DDIT3 VWA3A CST6 UCP3 IDSP1 SLCO3A1 C8orf31 SAMD9L JUN ODF3B ALS2CL EGR3 HCLS1 MUC16 HEPHL1 BAI1 UBA7 LINC00085 HCAR2 MX2 EMILIN3 KCNQ3 TACSTD2 FAM70B DUSP8 SOCS3 IFNE MAFF HEATR7B1 SOCS1 MAPK11 STAC3 MUC1 C11orf35 TLCD2 PDIA2 CYP4F12 RPS23 CEACAM19 MIR22HG EPOR MAGI2 DNAJB13 PALM3 HES4 NOXA1 APOD C6orf222 CXCL17 CGB7 HSH2D C5AR1 BCO2 CCDC154 SLC22A20 ZNF44 PLCG2 ARC GPRASP1 SNORA31 DDO AGER C6orf25 AC096670.3 IGFL4 GPR20 FAM221B LRRC10B FAM71F2 SAP25 ATP6AP1L CPT1B KRTAP5-1 LCAT LBH UCA1 PLIN5 RP11-83B20.1 RP11-429J17.8 RP11-1036E20.9 EVPLL SMTNL1 GCGR hsa-mir-6080 ATAD3C RP11-465B22.3 AC110619.2 SNORD63 ADM5 SH3BP5-AS1 RP11-288L9.4 NTF4 AC009950.2 RP11-203J24.8 RP11-69I8.3 RAB11FIP1P1 SAPCD1 RP4-583P15.10 AC019349.5 GOLGA6L5 RPS23P8 RP11-34A14.3 RP11-263K19.4 RP11-67C2.2 CTD-2020K17.3 RP11-250B2.3 SAPCD1-AS1 RP11-73M7.6 NRADDP CBR3-AS1 RNF223 RP3-430N8.8 AF011889.2 BX470102.3 APOBEC3G PSMB9 UGT1A9 RP11-147I3.1 RP11-379B18.5 AF011889.5 ARHGDIG Metazoa_SRP PDCL3P4 IFITM10 SCARF2 RP3-330M21.5 CTC-281B15.1 CTD-2248H3.1 RP11-510N19.5 CTC-325J23.2 CTD-2193P3.2 HMGB1P3 ALDH1L1-AS1 CTD-2193P3.1 CTC-459I6.1 RP11-459E5.1 CHKB-CPT1B RP11-496I9.1 RP11-326C3.2 MSH5-SAPCD1 RP11-167N4.2 RP11-512N21.3 RP11-631N16.2 RP11-386M24.3 MC1R RP4-647C14.3 RP11-566K11.4 RP11-521C20.3 RP11-1100L3.8 RP11-254F7.2 RP5-1085F17.3 RP11-386M24.6 AC145291.2 RP11-250B2.5 RP11-448G15.3 SPON1 LA16c-325D7.1 LINC00672 SNORD3A RP1-37N7.1 RP13-104F24.3 AC010761.10 RP5-906A24.2 MYO15B AC004510.3 SH3GL1P3 CTD-2240E14.4 CTD-3252C9.4 AC006262.5 CTD-2521M24.5
Down-regulated DEGs (287 genes)	CFTR SLC7A2 PLXND1 USH1C DPEP1 SLC38A5 GRAMD1B HSD17B6 MIPEP ZIC2 KITLG LTBP1 KCNQ1 GYG2 SOAT1 TMEM48 TBXAS1 SLC12A2 IPO5 PRR11 LIMS2 HMMR MCM2 PPP2R2C ENO1 PLD1 GPC4 EPB41L2 SLC4A4 SMARCA2 ABCB1 ORC1 IPO11 TPX2 TESC BIRC5 LYZ DPYSL2 TMEM38B BAMBI PCSK5 LGALS2 C14orf105 SERPINA4 MYBL2 HNF4A FERMT1 FGF9 SLC25A15 CPPED1 KIAA1199 MCM4 CDK6 CPVL COBL MOGAT3 AGR2 SLC1A1 AMBP DKK1 MAP2K6 ANXA10 GALNTL4 SOX6 POU2AF1 CHPT1 UST PERP PRLR HGD PRKAR2A ECT2 HHLA2 PLSCR4 IGFBP2 IL1R2 LEPR AGMAT NR5A2 PROX1 PLAGL1 AKAP7 MYB SLC16A7 GDA ONECUT2 MOB3B CCDC170 LYPLA1 RPL21 FAM126A TUBA1B EREG FOXA2 TMX4 AMOT WNK4 DLGAP5 SGPP1 TSPAN8 TUBA4A VIL1 GNAI1 MGAT3 HOXD13 DLL4 PPP1R1B NR0B2 DDC RFC3 ZDHHC8P1 HMGCS2 LDHA HNF1A AGT SLC19A3 SERPINE2 SMC2 HMGA1 CASC5 SLC38A4 LGR5 FAM222A SLC7A1 BRCA2 DIAPH3 ZIC5 NKD1 IMPA2 GATA6 PRKACB PARP1 LBR CHAC2 CDCA7 PTPRG CCNA2 FAM105A SYTL5 TACC1 LACTB2 FAM171A1 SLC43A1 ADRA2A C11orf53 SLC7A11 SLC2A13 SACS SPC25 CNKSR3 KCTD15 UBASH3B PLCL2 PPARGC1B SLC26A2 PTDSS1 HKDC1 ARSE ZNF618 EDA KALRN FGFR4 SSTR5 AKR7A3 CAMK2N1 PKDCC ARL5A KCNJ3 IHH FABP1 SAP30 MAD2L1 SPINK1 FABP5 HNF4G CLDN2 CDX2 KBTBD6 CKB ANPEP CCDC103 RRM1 FAM83B FEN1 NPNT CXXC4 SLC38A11 GJB1 ROBO1 P2RY1 MYO7B ALCAM SERPINA6 FOXA3 TMEM37 ESCO2 LGALS4 CYCS ADH6 AGR3 CSPG4 MUC13 FBXO45 KCNE3 KBTBD11 C18orf56 GSG2 LCN15 DNAJC22 KCTD12 TMEM64 OR51E1 C3orf58 C8orf33 EPHB3 NEB ASCL2 BRI3BP SFTPA2 TARSL2 GPRIN3 NCR3LG1 MAOA CTSE RP11-57C13.3 SERPINA1 CYP2B6 SPN DPP4 S100A10 PRIM1 AKR1B10 RYR2 PAPSS2 F5 SUCNR1 GRK5 C2orf72 SP5 C10orf112 ZBTB10 LGR4 LDHAP4 MUC5AC TENM3 AC007405.2 SLC25A5-AS1 NPY6R RP1-37C10.3 RP11-103C16.2 AC007163.6 RP11-229P13.23 COL4A2-AS1 AF196970.3 AC013463.2 MYB-AS1 AC005550.3 MLK7-AS1 RPS2P5 RP11-64D22.2 TDGF1 HNF1A-AS1 RP11-67L3.5 RP11-410D17.2 RP11-382J12.1 ADH1C SLC7A11-AS1 RP11-710F7.2 RP11-115D19.1 RAD21-AS1 PRKDC CTD-2292P10.2 RP11-363E6.3 RP11-700F16.3 RP11-627G23.1 RP11-173P15.3 TMPO-AS1 RP11-186F10.2 RP11-210N13.1 RP11-386G11.10 RP11-109D20.2 LINC00261 CTD-2196E14.5 RP11-401P9.4 CTD-2510F5.4 ZNF488 RP11-627G18.1

Supplementary Table 15. The topologically associating domains (TADs) with high intra-chromosomal interactions on chromosome 5.

chr1	x1	x2	chr2	y1	y2	score	uVarScore	lVarScore	upSign	loSign
chr5	80970000	81100000	chr5	80970000	81100000	0.634	0.047	0.058	0.599	0.522
chr5	81205000	81295000	chr5	81205000	81295000	0.944	0.017	0.035	0.633	0.722
chr5	81315000	81405000	chr5	81315000	81405000	0.853	0.038	0.006	0.700	0.611
chr5	81430000	81735000	chr5	81430000	81735000	0.701	0.094	0.103	0.593	0.503
chr5	81510000	81720000	chr5	81510000	81720000	0.982	0.045	0.080	0.803	0.639
chr5	81765000	81840000	chr5	81765000	81840000	0.776	0.051	0.144	0.609	0.516
chr5	82645000	82835000	chr5	82645000	82835000	1.389	0.084	0.113	0.647	0.884
chr5	82765000	82835000	chr5	82765000	82835000	0.888	0.016	0.169	0.518	0.607
chr5	82905000	83605000	chr5	82905000	83605000	0.219	0.435	0.341	0.411	0.419
chr5	87025000	87390000	chr5	87025000	87390000	1.347	0.314	0.319	0.581	0.655
chr5	90840000	91440000	chr5	90840000	91440000	0.737	0.354	0.392	0.414	0.439
chr5	94970000	95085000	chr5	94970000	95085000	1.354	0.075	0.119	0.688	0.521
chr5	95895000	96340000	chr5	95895000	96340000	1.020	0.343	0.290	0.498	0.535
chr5	96740000	97155000	chr5	96740000	97155000	1.037	0.327	0.308	0.402	0.537
chr5	100565000	101370000	chr5	100565000	101370000	0.851	0.378	0.316	0.457	0.406
chr5	102545000	103145000	chr5	102545000	103145000	1.206	0.332	0.337	0.537	0.484
chr5	102615000	103030000	chr5	102615000	103030000	0.924	0.331	0.312	0.423	0.451
chr5	111505000	112140000	chr5	111505000	112140000	1.311	0.323	0.323	0.466	0.660
chr5	112160000	112475000	chr5	112160000	112475000	1.194	0.294	0.331	0.651	0.417
chr5	116555000	116960000	chr5	116555000	116960000	0.665	0.271	0.290	0.410	0.459
chr5	118980000	119445000	chr5	118980000	119445000	0.730	0.350	0.408	0.474	0.483
chr5	120770000	121930000	chr5	120770000	121930000	0.675	0.366	0.394	0.401	0.468
chr5	128205000	128880000	chr5	128205000	128880000	0.979	0.367	0.318	0.439	0.536
chr5	130210000	130740000	chr5	130210000	130740000	0.673	0.357	0.339	0.408	0.413
chr5	136250000	136775000	chr5	136250000	136775000	0.507	0.321	0.421	0.403	0.460
chr5	136880000	137585000	chr5	136880000	137585000	0.615	0.321	0.307	0.476	0.432
chr5	137650000	137740000	chr5	137650000	137740000	1.380	0.106	0.090	0.567	0.656
chr5	146455000	146515000	chr5	146455000	146515000	0.985	0.045	0.131	0.500	0.500
chr5	154005000	154410000	chr5	154005000	154410000	0.873	0.370	0.355	0.484	0.478
chr5	174395000	174835000	chr5	174395000	174835000	0.967	0.369	0.339	0.411	0.425

Abbreviations
Score = corner score
Uvar = the variance of the upper triangle
Lvar = the variance of the lower triangle
Usign = -1*(sum of the sign of the entries in the upper triangle)
Lsign = sum of the sign of the entries in the lower triangle

Supplementary Table 16. The transportable elements of gene amplified region in MTX-resistant sample

Name	Class	Dir	Sim	Pos/Mm:Ts	Score
Gypsy-54_CGi-I	LTR/Gypsy	d	0.8333	3	244
Gypsy-9_DSer-I	LTR/Gypsy	d	0.7273	1.9091	220
LTR16C	ERV/ERV3	c	0.6718	1.7838	292
DIRS-25_XT	LTR/DIRS	d	0.7805	1.2857	233
L1-23_ATr	NonLTR/L1	d	0.8302	1.25	251
Alu2_TS	NonLTR/SINE/SINE1	d	0.6953	1.6765	357
Gypsy-8_ArHy-I	LTR/Gypsy	c	0.8788	2	229
Copia-7_DPu-I	LTR/Copia	d	0.7547	2.1667	220
Gypsy-70-I_NV	LTR/Gypsy	d	0.9032	1.5	233
Jockey-1_DBp	NonLTR/Jockey	d	0.7288	1.5	219
hAT-N27B_CPB	DNA/hAT	d	0.7692	1.5714	220
Gypsy-1_AIp-I	LTR/Gypsy	d	0.7872	1.2857	219
ALU	NonLTR/SINE/SINE1	c	0.7595	2.6667	261
MER1B	DNA/hAT	c	0.757	1.5641	1043
MIR	NonLTR/SINE/SINE2	c	0.76	2.125	316
Gypsy-18A_SB-I	LTR/Gypsy	c	0.8684	1	220
Copia-15_ECa-I	LTR/Copia	d	0.7746	2.4	214
DIRS-13_SpPu	LTR/DIRS	d	0.7297	1.5455	225
Tx1-7_Crp	NonLTR/Tx1	d	0.75	2	237
R2-1_PPap	NonLTR/R2	d	0.8043	1.1429	242
ALU	NonLTR/SINE/SINE1	c	0.7755	1.6	332
Copia-3_TMe-LTR	LTR/Copia	d	0.7424	1.4	238
BEL-673_AA-I	LTR/BEL	c	0.8788	4	210
Gypsy-40_PSt-I	LTR/Gypsy	c	0.7195	1.3846	213
Gypsy-14_ATr-LTR	LTR/Gypsy	d	0.688	1.5581	525
Gypsy-211_AA-I	LTR/Gypsy	d	0.766	1.4286	225
LTR-18F_OS-LTR	LTR/Gypsy	d	0.7103	1.3684	289
HELITRON2	DNA/Helitron	c	0.7042	1.1875	220
HAL1	NonLTR/L1	d	0.7727	1.2222	244
Gypsy-26_BiGl-I	LTR/Gypsy	c	0.7467	1.875	236
L1-15_STu	NonLTR/L1	d	0.8431	2	230
MuDR-9B_ALy	DNA/MuDR	c	0.7797	2.4	257
Clu-186_AG	DNA/Zator	c	0.7761	1.5714	236
Gypsy-34_SSa-I	LTR/Gypsy	c	0.9	1	222
MUDRB_PT	DNA/MuDR	c	0.9286	1	230
Dada-3_PMar	DNA/Dada	c	0.766	1.4286	223
Gypsy4-NVi_LTR	LTR/Gypsy	d	0.8049	1.1667	232
MuDR-2_BRa	DNA/MuDR	c	0.8788	2	221
RTE-41_LMi	NonLTR/RTE	d	0.8085	2	239
L1-2_Cja	NonLTR/L1	d	0.8689	1.5	298
Harbinger-1_AAe	DNA/Harbinger	c	0.8485	1	226
Daphne-17_LMi	NonLTR/Daphne	c	0.8182	1.75	221
Gypsy-8_PIT-I	LTR/Gypsy	c	0.82	3.5	227
THE1B	ERV/ERV3	c	0.7678	1.4865	940
L1-2_ALy	NonLTR/L1	c	0.825	1.4	242
L1ME3C_3end	NonLTR/L1	d	0.6818	1.6145	756

Name: locus names of submitted query sequences;Dir: orientation ('d' for direct, 'c' for complementary) of repeat fragment;Sim: value of similarity between 2 aligned fragments;Pos: the ratio of positives to alignment length;Score: the alignment score obtained from blast;Class: class/subclass of repeat as specified in repeat annotation;Mm:Ts : a ratio of mismatches to transitions in nucleotide alignment

Supplementary Data 1. The filtered variants and CNV category in control

chrom1	pos1	strand1	chrom2	pos2	strand2	variant_name	variant_type	split	size	CNV_category	category	nearby_variant_count
2	96593164	+	2	96593207	+	5	INVDUP	23	43	neutral	simple	0
2	164156945	+	2	164157034	+	6	INVDUP	61	89	neutral	simple	0
2	190973837	+	2	190973945	+	7	INVDUP	12	108	neutral	simple	0
3	98691865	+	3	98695939	-	10	DEL	10	4074	neutral	simple	0
3	162794412	+	3	162908536	-	13	DEL	10	114124	matching	simple	0
5	1178179	+	5	1180610	-	17	DEL	14	2431	neutral	simple	0
5	84652556	+	5	84658976	-	19	DEL	10	6420	neutral	simple	0
6	28371976	-	6	28372014	-	21	INVDUP	47	38	neutral	simple	0
7	107625087	+	7	107625138	+	23	INVDUP	15	51	non-matching	simple	0
8	125582886	+	8	125588887	-	40	DEL	34	6001	neutral	simple	0
8	126179966	+	8	126182327	-	44	DEL	15	2361	partial	simple	0
8	134070687	+	8	134076773	-	53	DEL	20	6086	neutral	simple	0
8	141492229	-	8	141493233	+	72	DUP	11	1004	neutral	simple	0
9	89561164	+	9	89563024	-	76	DEL	10	1860	neutral	simple	0
9	93708074	+	9	93709182	-	77	DEL	10	1108	neutral	simple	0
10	112352425	+	10	112356897	-	79	DEL	11	4472	neutral	simple	0
11	123742618	+	11	123742654	+	82	INVDUP	11	36	neutral	simple	0
13	29641713	+	13	29647708	-	89	DEL	12	5995	neutral	simple	0
16	65573316	+	16	65573355	+	101	INVDUP	18	39	neutral	simple	0
17	32750176	+	17	32794396	-	102	DEL	14	44220	neutral	simple	0
18	54426598	+	18	54430590	-	105	DEL	10	3992	neutral	simple	0
22	29476739	-	22	29476879	-	110	INVDUP	10	140	neutral	simple	0
1	159657699	-	18	5830429	-	145	TRA	31	-1	neutral	solo	0
2	32867129	+	12	66057593	-	146	TRA	68	-1	neutral	reciprocal	16
2	32866714	-	7	107770275	+	147	TRA	93	-1	neutral	reciprocal	11
2	32867129	+	20	34228129	-	148	TRA	15	-1	neutral	crowded	9
2	32867129	+	15	54926030	-	149	TRA	15	-1	neutral	reciprocal	9
3	10000	-	5	11888	+	150	TRA	12	-1	neutral	reciprocal	2
3	10984	+	5	10001	-	151	TRA	12	-1	neutral	reciprocal	2
3	93470361	-	8	43236961	-	152	TRA	17	-1	neutral	reciprocal	9
3	93470361	-	4	49709071	-	153	TRA	11	-1	partial	reciprocal	11
3	93470361	-	14	16096603	+	155	TRA	42	-1	partial	reciprocal	13
3	93470361	-	22	11210996	-	156	TRA	11	-1	neutral	reciprocal	13
3	93470801	+	14	16091757	-	158	TRA	22	-1	non-matching	reciprocal	13
3	93470811	+	22	11215576	+	160	TRA	12	-1	neutral	reciprocal	13
3	93470800	+	4	49711963	+	161	TRA	13	-1	non-matching	reciprocal	11
3	93470814	+	8	43242402	+	163	TRA	12	-1	neutral	reciprocal	9
3	111555239	-	8	127521310	-	164	TRA	17	-1	neutral	solo	0
3	198174528	+	5	11888	+	165	TRA	14	-1	partial	crowded	2
4	49709071	-	22	11215532	+	166	TRA	22	-1	partial	reciprocal	10
4	49709072	-	14	16091759	-	169	TRA	16	-1	partial	crowded	10
4	49711964	+	22	11210968	-	170	TRA	22	-1	non-matching	reciprocal	10
4	81594198	-	12	66057683	+	172	TRA	13	-1	neutral	crowded	11
4	130425242	+	12	66057684	+	173	TRA	12	-1	neutral	crowded	11
5	88112381	+	12	66057683	+	174	TRA	25	-1	neutral	crowded	11
6	156846465	-	X	20815784	+	175	TRA	18	-1	neutral	solo	0
7	107769994	-	12	66057683	+	176	TRA	54	-1	neutral	reciprocal	16
7	107770275	+	15	54926030	-	177	TRA	23	-1	neutral	crowded	10
7	107770275	+	20	34228129	-	178	TRA	23	-1	neutral	crowded	9
7	107770275	+	X	79200470	+	179	TRA	15	-1	neutral	crowded	6
7	116455218	+	11	98184802	+	180	TRA	17	-1	neutral	crowded	1
8	43242443	+	14	16096599	+	181	TRA	11	-1	partial	crowded	8
11	98184607	-	20	13660993	+	182	TRA	17	-1	neutral	crowded	1
12	66057593	-	20	34228266	+	183	TRA	13	-1	neutral	reciprocal	13
12	66057592	-	15	54926085	+	184	TRA	49	-1	neutral	reciprocal	14
12	66057592	-	16	81086163	-	185	TRA	12	-1	neutral	crowded	11
13	108139825	+	X	127952807	+	186	TRA	11	-1	neutral	solo	0
14	16091765	-	22	11210951	-	187	TRA	16	-1	non-matching	reciprocal	11
2	32866714	-	12	66057683	+	196	TRA	63	-1	neutral	reciprocal	16
2	32867129	+	7	107769994	-	197	TRA	51	-1	neutral	reciprocal	11
2	32866714	-	15	54926085	+	198	TRA	12	-1	neutral	reciprocal	9
3	93470800	+	22	11215532	+	199	TRA	17	-1	neutral	reciprocal	13
7	107770275	+	12	66057593	-	200	TRA	120	-1	neutral	reciprocal	16
12	66057683	+	20	34228129	-	201	TRA	33	-1	neutral	reciprocal	13
12	66057683	+	15	54926030	-	202	TRA	31	-1	neutral	reciprocal	14
14	16091758	-	22	11211013	-	203	TRA	19	-1	non-matching	reciprocal	11
14	16096548	+	22	11215541	+	204	TRA	11	-1	partial	reciprocal	11

Supplementary Data 2. The filtered variants and CNV category in MTX resistant HT-29

chrom1	pos1	strand1	chrom2	pos2	strand2	variant_name	variant_type	split	size	CNV_category	category	nearby_variant_count
1	16725232	-	1	234776440	-	0	INV	11	218051208	non-matching	solo	0
1	192559133	+	1	192559190	+	2	INVDUP	11	57	neutral	simple	0
1	194482045	+	1	194484971	-	3	DEL	11	2926	neutral	simple	0
2	86669095	-	2	86669309	-	12	INVDUP	16	214	neutral	simple	0
2	32866714	-	7	107770275	+	226	TRA	126	-1	partial	reciprocal	17
2	32867129	+	12	66057593	-	227	TRA	89	-1	non-matching	reciprocal	22
2	32866714	+	4	130425242	+	228	TRA	17	-1	non-matching	crowded	12
2	32866714	+	18	63063646	-	229	TRA	15	-1	non-matching	crowded	11
2	32866714	-	15	54926085	+	230	TRA	18	-1	partial	reciprocal	14
2	32867111	+	4	49273782	+	231	TRA	12	-1	partial	crowded	13
2	32867129	+	10	109812363	-	232	TRA	14	-1	non-matching	crowded	11
2	32867129	+	20	34228129	-	233	TRA	32	-1	non-matching	reciprocal	14
2	32867129	+	7	107769994	-	277	TRA	67	-1	non-matching	reciprocal	17
2	32866714	-	12	66057683	+	278	TRA	76	-1	partial	reciprocal	22
2	32867129	+	15	54926030	-	279	TRA	13	-1	non-matching	reciprocal	14
2	32866714	-	20	34228294	+	280	TRA	13	-1	partial	reciprocal	14
3	93470361	-	22	11211022	-	234	TRA	26	-1	neutral	reciprocal	16
3	93470361	-	4	49709071	-	237	TRA	15	-1	partial	reciprocal	13
3	93470361	-	8	43236971	-	238	TRA	15	-1	non-matching	reciprocal	12
3	93470361	-	14	16096600	+	239	TRA	27	-1	partial	reciprocal	16
3	93470800	+	4	49711967	+	240	TRA	25	-1	non-matching	reciprocal	13
3	93470800	+	8	43242388	+	242	TRA	12	-1	partial	reciprocal	12
3	93470800	+	22	11215540	+	244	TRA	49	-1	neutral	reciprocal	16
3	93470800	+	14	16091765	-	245	TRA	30	-1	non-matching	reciprocal	16
3	111555239	-	8	127521400	-	246	TRA	20	-1	neutral	solo	0
3	93470361	-	22	11210950	-	281	TRA	16	-1	neutral	reciprocal	16
3	93470807	+	14	16091707	-	283	TRA	18	-1	non-matching	reciprocal	16
4	49273694	+	12	66057683	+	247	TRA	13	-1	partial	crowded	14
4	49273782	+	7	107770275	+	248	TRA	12	-1	partial	crowded	9
4	49709071	-	14	16091787	-	250	TRA	19	-1	partial	reciprocal	13
4	49709071	-	22	11215532	+	251	TRA	15	-1	partial	reciprocal	13
4	49711966	+	14	16096635	+	252	TRA	12	-1	partial	reciprocal	13
4	49711969	+	22	11211007	-	253	TRA	19	-1	non-matching	reciprocal	13
4	130425242	+	12	66057684	+	255	TRA	25	-1	neutral	crowded	13
5	80617089	-	5	80618750	-	24	INVDUP	47	1661	matching	simple	0
5	81781592	+	5	81781648	+	50	INVDUP	10	56	neutral	simple	0
5	82245207	+	5	82573904	+	56	INV	56	328697	matching	crowded	1
5	82470789	-	5	82842464	+	58	DUP	11	371675	matching	crowded	4
5	82470887	+	5	82804501	+	59	INV	12	333614	partial	crowded	4
5	82473031	-	5	82474560	-	60	INVDUP	18	1529	matching	simple	3
5	82678370	+	5	82764579	-	62	DEL	56	86209	matching	simple	3
5	82804502	+	5	82842464	+	64	INV	47	37962	matching	simple	3
5	137519108	+	5	137520622	-	65	DEL	19	1514	neutral	simple	0
5	137686889	+	5	137688197	-	66	DEL	22	1308	neutral	simple	0
5	88112313	-	12	66057592	-	256	TRA	12	-1	neutral	reciprocal	12
5	88112381	+	12	66057683	+	284	TRA	12	-1	neutral	reciprocal	12
6	119350199	-	22	21808762	+	257	TRA	12	-1	neutral	solo	0
6	160100725	+	12	66057638	-	258	TRA	12	-1	neutral	crowded	12

The first 50 lines of supplementary Data 2 were shown in table.

Supplementary Data 3. The comparison of detected somatic copy number aberrations between control and MTX resistant HT-29

CHROM	Position (start)	Position (end)	Control	MTX resistant HT-29	Log2ratio
**chr1**	0	10000	0.0019	0.0019	0
**chr1**	10000	20000	15.268	11.2173	-0.444785627
**chr1**	20000	30000	10.1306	6.7845	-0.578405214
**chr1**	30000	40000	1.9296	2.1866	0.180387508
**chr1**	40000	50000	2.6358	2.655	0.010470955
**chr1**	50000	60000	4.6441	2.443	-0.926745146
**chr1**	60000	70000	6.7541	2.4643	-1.454585604
**chr1**	70000	80000	1.8279	1.2424	-0.557057374
**chr1**	80000	90000	2.464	1.0061	-1.292228464
**chr1**	90000	100000	3.7317	1.8558	-1.007791731
**chr1**	100000	110000	7.5037	2.8784	-1.382335027
**chr1**	110000	120000	7.5825	7.4175	-0.031740571
**chr1**	120000	130000	8.429	3.3052	-1.350623883
**chr1**	130000	140000	3.9159	1.6947	-1.208313969
**chr1**	140000	150000	0.604	1.3235	1.13173761
**chr1**	150000	160000	1.2211	1.0336	-0.240503356
**chr1**	160000	170000	1.2039	1.0364	-0.216134622
**chr1**	170000	180000	3.9674	6.0622	0.611647577
**chr1**	180000	190000	43.4247	35.8975	-0.274632507
**chr1**	190000	200000	13.5297	12.8423	-0.075226244
**chr1**	200000	210000	2.9472	1.4439	-1.029374086
**chr1**	210000	220000	0	0	0
**chr1**	220000	230000	0	0	0
**chr1**	230000	240000	0	0	0
**chr1**	240000	250000	0	0	0
**chr1**	250000	260000	0.6622	1.6236	1.2938572
**chr1**	260000	270000	5.516	8.4993	0.62372156
**chr1**	270000	280000	10.6417	7.7842	-0.45110795
**chr1**	280000	290000	6.1073	6.9128	0.178735469
**chr1**	290000	300000	2.2304	1.5081	-0.56457034
**chr1**	300000	310000	0	0	0
**chr1**	310000	320000	0	0	0
**chr1**	320000	330000	0	0	0
**chr1**	330000	340000	0	0	0
**chr1**	340000	350000	0	0	0
**chr1**	350000	360000	3.3039	1.9593	-0.753831677
**chr1**	360000	370000	2.4763	2.5228	0.026839732
**chr1**	370000	380000	3.5476	4.0496	0.190936056
**chr1**	380000	390000	4.2914	3.7582	-0.191406531
**chr1**	390000	400000	5.607	3.6191	-0.631598084
**chr1**	400000	410000	6.3723	1.8939	-1.750453978
**chr1**	410000	420000	6.5152	3.4431	-0.920101364
**chr1**	420000	430000	9.0299	3.7597	-1.26409244
**chr1**	430000	440000	4.4141	3.4593	-0.351639171
**chr1**	440000	450000	2.564	6.4229	1.324828539
**chr1**	450000	460000	3.2647	2.9273	-0.157379815
**chr1**	460000	470000	6.7922	1.2193	-2.477825709

The first 50 lines of supplementary Data 3 were shown in table.

Supplementary Data 4. The detected mutations in MTX resistant HT-29

Chr	Start	End	Ref	Alt	Func.refGene	Gene.refGene
chr1	14522	14522	G	A	ncRNA_exonic	WASH7P
chr1	14542	14542	A	G	ncRNA_exonic	WASH7P
chr1	14574	14574	A	G	ncRNA_exonic	WASH7P
chr1	14590	14590	G	A	ncRNA_exonic	WASH7P
chr1	14599	14599	T	A	ncRNA_exonic	WASH7P
chr1	14604	14604	A	G	ncRNA_exonic	WASH7P
chr1	14610	14610	T	C	ncRNA_exonic	WASH7P
chr1	14653	14653	C	T	ncRNA_exonic	WASH7P
chr1	14907	14907	A	G	ncRNA_intronic	WASH7P
chr1	14930	14930	A	G	ncRNA_intronic	WASH7P
chr1	15118	15118	A	G	ncRNA_intronic	WASH7P
chr1	15211	15211	T	G	ncRNA_intronic	WASH7P
chr1	15274	15274	A	T	ncRNA_intronic	WASH7P
chr1	15447	15447	A	G	ncRNA_intronic	WASH7P
chr1	15903	15903	-	C	ncRNA_exonic	WASH7P
chr1	15906	15906	A	G	ncRNA_exonic	WASH7P
chr1	16014	16014	C	T	ncRNA_intronic	WASH7P
chr1	16068	16068	T	C	ncRNA_intronic	WASH7P
chr1	16125	16125	T	G	ncRNA_intronic	WASH7P
chr1	16257	16257	G	C	ncRNA_intronic	WASH7P
chr1	16298	16298	C	T	ncRNA_intronic	WASH7P
chr1	16378	16378	T	C	ncRNA_intronic	WASH7P
chr1	16495	16495	G	C	ncRNA_intronic	WASH7P
chr1	16534	16534	C	T	ncRNA_intronic	WASH7P
chr1	16949	16949	A	C	ncRNA_exonic	WASH7P
chr1	17020	17020	G	A	ncRNA_exonic	WASH7P
chr1	17375	17375	A	G	ncRNA_exonic	MIR6859-1;MIR6859-2;MIR6859-3;MIR6859-4
chr1	17385	17385	G	A	ncRNA_exonic	MIR6859-1;MIR6859-2;MIR6859-3;MIR6859-4
chr1	17406	17406	C	T	ncRNA_exonic	MIR6859-1;MIR6859-2;MIR6859-3;MIR6859-4
chr1	17594	17594	C	T	ncRNA_intronic	WASH7P
chr1	18353	18353	C	T	ncRNA_exonic	WASH7P
chr1	19217	19217	G	C	ncRNA_intronic	WASH7P
chr1	19322	19322	C	T	ncRNA_intronic	WASH7P
chr1	19355	19355	G	A	ncRNA_intronic	WASH7P
chr1	19391	19391	G	A	ncRNA_intronic	WASH7P
chr1	20129	20129	C	T	ncRNA_intronic	WASH7P
chr1	20166	20166	A	G	ncRNA_intronic	WASH7P
chr1	20191	20191	T	G	ncRNA_intronic	WASH7P
chr1	20207	20207	G	A	ncRNA_intronic	WASH7P
chr1	20212	20212	A	G	ncRNA_intronic	WASH7P
chr1	20235	20235	G	A	ncRNA_intronic	WASH7P
chr1	20392	20392	G	A	ncRNA_intronic	WASH7P
chr1	21503	21503	G	T	ncRNA_intronic	WASH7P
chr1	22011	22011	G	A	ncRNA_intronic	WASH7P
chr1	22012	22012	A	G	ncRNA_intronic	WASH7P
chr1	22113	22113	T	G	ncRNA_intronic	WASH7P
chr1	24786	24786	G	A	ncRNA_exonic	WASH7P
chr1	26161	26161	T	A	ncRNA_intronic	WASH7P

The first 50 lines of supplementary Data 4 were shown in table.

Supplementary Data 5. The detected mutations in control

Chr	Start	End	Ref	Alt	Func.refGene	Gene.refGene
chr1	12948	12948	T	C	ncRNA_intronic	DDX11L1
chr1	14522	14522	G	A	ncRNA_exonic	WASH7P
chr1	14542	14542	A	G	ncRNA_exonic	WASH7P
chr1	14574	14574	A	G	ncRNA_exonic	WASH7P
chr1	14590	14590	G	A	ncRNA_exonic	WASH7P
chr1	14599	14599	T	A	ncRNA_exonic	WASH7P
chr1	14604	14604	A	G	ncRNA_exonic	WASH7P
chr1	14610	14610	T	C	ncRNA_exonic	WASH7P
chr1	14653	14653	C	T	ncRNA_exonic	WASH7P
chr1	15118	15118	A	G	ncRNA_intronic	WASH7P
chr1	15274	15274	A	T	ncRNA_intronic	WASH7P
chr1	16257	16257	G	C	ncRNA_intronic	WASH7P
chr1	16298	16298	C	T	ncRNA_intronic	WASH7P
chr1	16378	16378	T	C	ncRNA_intronic	WASH7P
chr1	16495	16495	G	C	ncRNA_intronic	WASH7P
chr1	16534	16534	C	T	ncRNA_intronic	WASH7P
chr1	16949	16949	A	C	ncRNA_exonic	WASH7P
chr1	17385	17385	G	A	ncRNA_exonic	MIR6859-1;MIR6859-2;MIR6859-3;MIR6859-4
chr1	17728	17728	G	T	ncRNA_exonic	WASH7P
chr1	19217	19217	G	C	ncRNA_intronic	WASH7P
chr1	19226	19226	T	A	ncRNA_intronic	WASH7P
chr1	19249	19249	G	A	ncRNA_intronic	WASH7P
chr1	19254	19254	G	C	ncRNA_intronic	WASH7P
chr1	19279	19279	C	T	ncRNA_intronic	WASH7P
chr1	19322	19322	C	T	ncRNA_intronic	WASH7P
chr1	19355	19355	G	A	ncRNA_intronic	WASH7P
chr1	19391	19391	G	A	ncRNA_intronic	WASH7P
chr1	19425	19425	A	G	ncRNA_intronic	WASH7P
chr1	19432	19432	A	G	ncRNA_intronic	WASH7P
chr1	20129	20129	C	T	ncRNA_intronic	WASH7P
chr1	20166	20166	A	G	ncRNA_intronic	WASH7P
chr1	21419	21419	C	T	ncRNA_intronic	WASH7P
chr1	21580	21580	C	T	ncRNA_intronic	WASH7P
chr1	22018	22018	T	C	ncRNA_intronic	WASH7P
chr1	23975	23975	G	A	ncRNA_intronic	WASH7P
chr1	28511	28511	G	A	ncRNA_intronic	WASH7P
chr1	28558	28558	C	T	ncRNA_intronic	WASH7P
chr1	28563	28563	A	G	ncRNA_intronic	WASH7P
chr1	28589	28589	-	TTGG	ncRNA_intronic	WASH7P
chr1	118617	118617	T	C	intergenic	OR4F5;LOC729737
chr1	122872	122872	T	G	intergenic	OR4F5;LOC729737
chr1	127491	127491	T	G	intergenic	OR4F5;LOC729737
chr1	127573	127573	G	A	intergenic	OR4F5;LOC729737
chr1	129285	129285	G	A	intergenic	OR4F5;LOC729737
chr1	135982	135982	A	G	ncRNA_exonic	LOC729737
chr1	136048	136048	C	T	ncRNA_exonic	LOC729737
chr1	136052	136052	T	C	ncRNA_exonic	LOC729737
chr1	136145	136145	T	C	ncRNA_exonic	LOC729737

The first 50 lines of supplementary Data 5 were shown in table.
